# Disulfidptosis and Its Hub Gene Slc3a2 Involved in Ulcerative Colitis Pathogenesis, Disease Progression, and Patient Responses to Biologic Therapies

**DOI:** 10.3390/ijms252413506

**Published:** 2024-12-17

**Authors:** Qing-Qing Yang, Jun-An Guo, Ke Zhang, Si-Hui Li, Wan-Yu Xia, De-Xian Wang, Lu-Shuang Xie, Jun-Meng Wang, Qiao-Feng Wu

**Affiliations:** 1 Acupuncture and Moxibustion School, Chengdu University of Traditional Chinese Medicine, Chengdu 610075, China; 2 College of Intelligent Medicine, Chengdu University of Traditional Chinese Medicine, Chengdu 610075, China; 3 College of Basic Medicine, Chengdu University of Traditional Chinese Medicine, Chengdu 610075, China; 4 Key Laboratory of Acupuncture for Senile Disease (Chengdu University of Traditional Chinese Medicine), Ministry of Education, Chengdu 610075, China; 5 Institute of Acupuncture and Homeostasis Regulation, Chengdu University of Traditional Chinese Medicine, Chengdu 610075, China

**Keywords:** ceRNA network, disulfidptosis, integrated analysis, machine learning, ulcerative colitis, small molecule agents prediction, SLC3A2

## Abstract

To analyze the role of disulfidptosis in ulcerative colitis (UC), large-scale datasets combined with weighted gene co-expression network analysis (WGCNA) and machine learning were utilized and analyzed. When the hub genes that are associated with UC disease phenotypes and have predictive performance were identified, immune cell infiltration and the CeRNA network were constructed, the role of hub genes in UC pathogenies and biotherapy were investigated, and molecular docking studies and mice-verified tests were carried out to further explore the potential core genes and potential target. Finally, we found 21 DRGs involved in UC pathogenesis, including SLC3A2, FLNA, CAPZB, TLN1, RPN1, etc. Moreover, SLC3A2, TLN1, and RPN1 show a notable correlation with UC inflammatory state, and the expression of DRGs is closely related to the response to UC biotherapy. Our study suggests that disulfidptosis plays a crucial role in the pathogenesis and disease progression of UC. Higher expression of DRGs is commonly observed in moderate to severe UC patients, which may also affect their response to biologic therapies. Among the identified genes, SLC3A2 stands out, providing new insights into the underlying mechanisms of UC and potentially serving as a novel therapeutic target for the treatment of UC.

## 1. Introduction

Ulcerative colitis (UC) is an increasingly severe inflammatory bowel disease (IBD) characterized by chronic idiopathic inflammation primarily affecting the colonic mucosa and submucosa. Despite progress in diagnosis and treatment, the management of UC remains challenging due to limited knowledge of its pathogenesis and mechanisms [1,2,3]. Therefore, studying the molecular mechanisms of UC is crucial for improving prevention, diagnosis, and treatment strategies. During the occurrence of UC, damage to the intestinal mucosa can lead to nutrient absorption disorders and chronic diarrhea, resulting in a relative glucose deficiency. This glucose deficiency can impair normal metabolic processes, including the pentose phosphate pathway (PPP), which is a key pathway for NADPH production [4,5,6]. NADPH plays an important reducing role in cells, including participating in the formation of disulfide bonds and maintaining cellular redox balance. Specifically, glucose is converted into pyruvate during glycolysis, while NADPH is also produced. NADPH, as an important reducing agent, participates in various biosynthetic reactions, including fatty acid synthesis, cholesterol synthesis, and protein disulfide bond formation [7]. When NADPH is depleted, it can lead to the accumulation and stress of excessive disulfides, such as disulfides, thereby exacerbating the occurrence of cell death [8]. This form of cell death caused by the accumulation of intracellular disulfides is called disulfidoptosis. Elevated levels of cysteine and cysteine/cysteine ratio were observed in UC tissues, indicating the accumulation of disulfides in the intestinal mucosa [9].

Thiol antioxidants have been shown to significantly inhibit oxidative reactions induced by TNBS/LPS, thereby preventing associated cellular damage and cytokine secretion. This protective effect extends to reducing colonic and renal injury in mice [10]. Furthermore, *Bifidobacterium dentium* can secrete γ-glutamylcysteine, an intermediate in glutathione synthesis, which enhances glutathione levels, reduces reactive oxygen species (ROS) generated by endoplasmic reticulum (ER) stress, and inhibits the activation of the NF-kB pathway. This mechanism alleviates ER stress and increases the expression of MUC2 and IL-10 in the colon, thereby helping to restore the intestinal mucus barrier and alleviate colitis [11]. Therefore, the symptom relief observed in UC treated with thiol antioxidants may be linked to the oxidative stress of disulfidptosis or reducing disulfide bond formation. By lowering the oxidative burden, thiol antioxidants can help restore redox balance and improve cellular function in the intestinal mucosa, contributing to the overall management of UC symptoms. Thus, it is essential to elucidate the relationship between disulfidoptosis and UC and explore its role and regulatory mechanisms in UC intestinal mucosa.

In this study, we integrated large-scale datasets, weighted gene co-expression network analysis (WGCNA), and machine learning to clearly define the important role of disulfidoptosis-related genes (DRGs) in the pathogenesis of UC. We validated the identified key genes including SLC3A2, FLNA, CAPZB, TLN1, and RPN1 through symptom-related datasets and drug response-related datasets, elucidating the involvement of DRGs in disease progression and drug response. Our findings indicate that there are widespread aberrant expressions of DRGs in UC, with key DRGs showing a close relationship with immune cells. The lack of drug response in moderate to severe UC patients may be related to the increased expression of DRGs. Additionally, small-molecule drug predictions have provided us with new insights into the pathogenesis of ulcerative colitis, particularly targeting the abnormal activation of the sympathetic nervous system, with SLC3A2 potentially serving as a new molecular target.

The datasets GSE179285 and GSE206285 were used as training sets to identify differentially expressed genes (DEGs) between healthy individuals and patients with ulcerative colitis (UC). Weighted gene co-expression network analysis (WGCNA) was performed to construct the co-expression network and to screen for module genes. The intersection of disulfidptosis-related genes (DRGs), DEGs, and module genes led to the identification of characteristic genes. Least absolute shrinkage and selection operator (LASSO) and support vector machine (SVM) algorithms were applied to pinpoint hub genes. The significance of these hub genes in UC was validated by assessing their expression patterns, correlation with symptoms, and responsiveness to biological agents across some external validation sets (GSE48958, GSE47908, GSE92415, GSE75214, GSE87466, GSE92415, and GSE73661). Immunoinfiltration and correlation analyses were subsequently conducted to explore the relationships between hub genes and immune cells. Finally, small molecule agents were screened, and a competing endogenous RNA (CeRNA) regulation network associated with disulfidptosis was constructed. Notably, we used the DSS-induced colitis model to validate our findings.

## 2. Results

### 2.1. Significant Differential Expression of DRGs in UC

**Figure 1:**
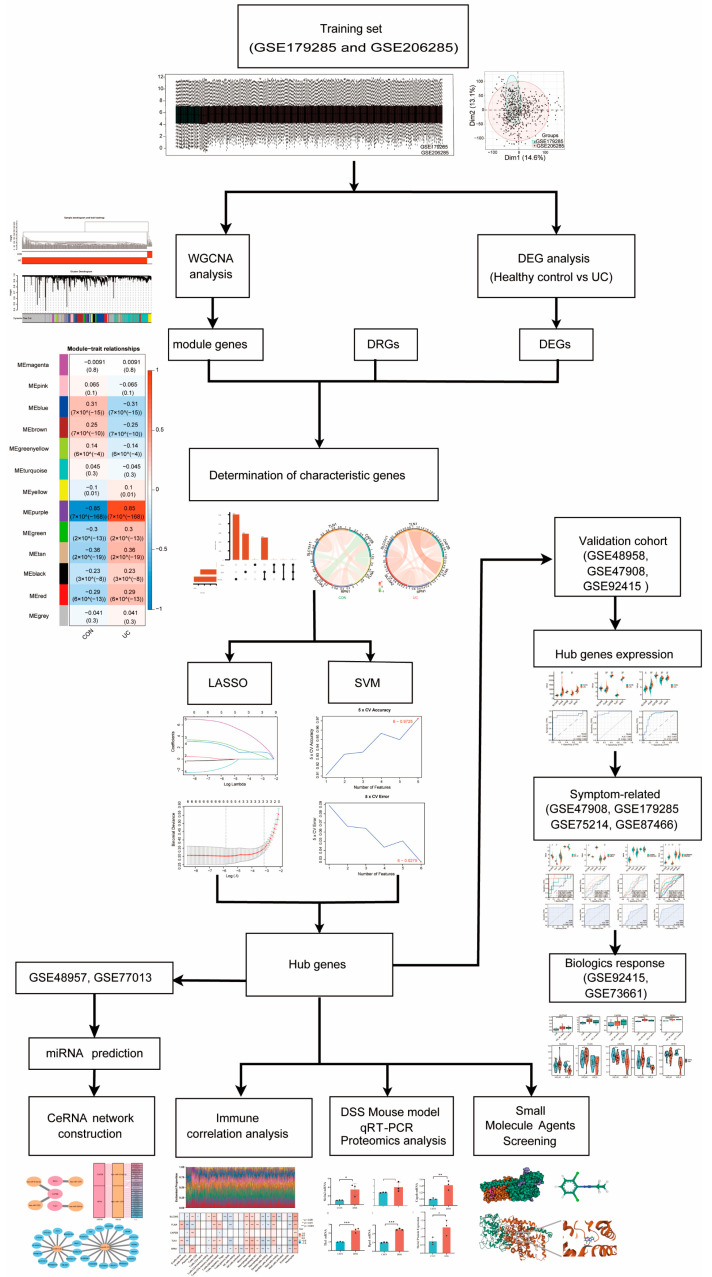
Study flowchart. The datasets GSE179285 and GSE206285 were used as training sets to identify differentially expressed genes (DEGs) between healthy individuals and patients with ulcerative colitis (UC). Weighted gene co-expression network analysis (WGCNA) was performed to construct the co-expression network and to screen for module genes. The intersection of disulfidptosis-related genes (DRGs), DEGs, and module genes led to the identification of characteristic genes. Least absolute shrinkage and selection operator (LASSO) and support vector machine (SVM) algorithms were applied to pinpoint hub genes. The significance of these hub genes in UC was validated by assessing their expression patterns, correlation with symptoms, and responsiveness to biological agents across seven external validation sets (GSE48958, GSE47908, GSE92415, GSE75214, GSE87466, GSE92415, and GSE73661). Immunoinfiltration and correlation analyses were subsequently conducted to explore the relationships between hub genes and immune cells. Finally, small molecule agents were screened, and a competing endogenous RNA (CeRNA) regulation network associated with disulfidptosis was constructed. By the way we used the DSS-induced colitis model to validate our findings.

**Figure 2:**
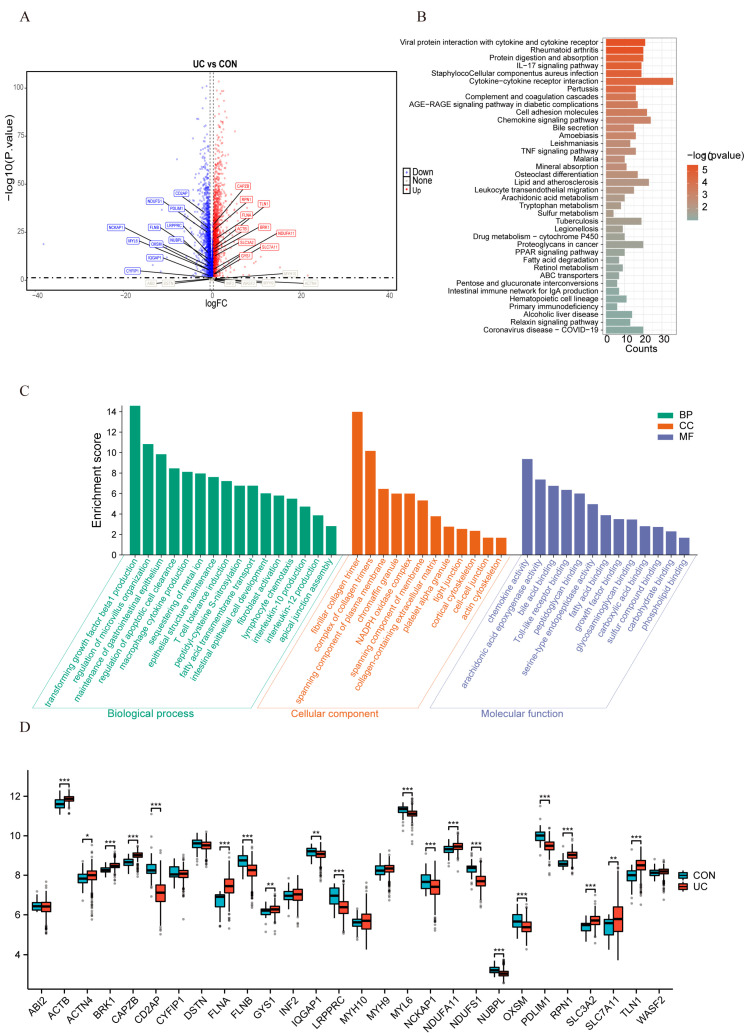
Integrated datasets analysis and differential expression of DRGs in UC. (**A**) The blue dots represent genes that are significantly downregulated in the disease state with an adjusted *p*-value less than 0.05 and a fold change (FC) less than −1.2. The red dots represent genes that are significantly upregulated in the disease state with an adjusted *p*-value less than 0.05 and an FC greater than 1.2. The gray dots represent genes with no statistically significant difference in expression between the disease and control states. (**B**) Kyoto Encyclopedia of Genes and Genomes (KEGG) pathway enrichment analysis of the top 360 DEGs. (**C**) Gene Ontology (GO) functional enrichment analysis, including Biological Processes (BP), Cellular Components (CC), and Molecular Functions (MF), revealing the underlying functions of the top 360 DEGs. (**D**) Box plot illustrating the expression differences in DRGs between healthy individuals and UC patients. The boxes represent the interquartile range (IQR), with the line inside the box indicating the median expression level. * *p* < 0.05, ** *p* < 0.01, *** *p* < 0.001.

The flow chart of this study is illustrated in Figure 1. To investigate the expression of DRGs in the intestinal mucosa of UC patients, we downloaded the GSE179285 and GSE206285 datasets from the GEO database. After performing batch effect correction, our training set included 563 UC samples and 49 healthy control samples. Genes with an adjusted *p*-value < 0.05 and a |log2 (fold change)| ≥ log2 (1.2) were considered statistically significant, resulting in the identification of 3061 upregulated genes and 2303 downregulated genes (Figure 2A and Table S3). Notably, we highlighted the position of DRGs within the set of DEGs, underscoring their relevance in the context of UC.

We performed KEGG and GO functional enrichment analyses to further characterize the biological features of the DEGs. The results of the KEGG analysis revealed several significantly enriched pathways, including “Cytokine-cytokine receptor interaction”, “Rheumatoid arthritis”, and “Intestinal immune network for IgA production” (Figure 2B). These pathways are well-established in the context of UC pathogenesis, reinforcing the robustness of our dataset integration.

In addition, the GO enrichment analysis highlighted key processes such as “maintenance of gastrointestinal epithelium”, “regulation of apoptotic cell clearance”, “T cell tolerance induction”, and “interleukin-10 production” in Biological Processes (BP). In Cellular Components (CC), there are identified terms like “NADPH oxidase complex”, “tight junction”, and “actin cytoskeleton”. Molecular Functions (MF) identified significant enrichments in “Toll-like receptor binding”, “glycosaminoglycan binding”, and “sulfur compound binding” (Figure 2C), further illustrating that the pathology of UC is closely associated with sulfur compound synthesis, cytoskeletal-related functions, and NADPH, while also highlighting its immune-related characteristics.

Our analysis of DRG expression in UC patients compared to healthy controls revealed significant differential expression of DRGs. Specifically, SLC3A2, SLC7A11, ACTN4, CAPZB, FLNA, GYS1, NDUFA11, RPN1, and TLN1 were significantly upregulated in the intestinal mucosa of UC patients, while CD2AP, FLNB, IQGAP1, LRPPRC, MYL6, NCKAP1, NDUFS1, NUBPL, OXSM, and PDLIM1 were downregulated (Figure 2D). These marked expression differences not only underscore the dysregulation of DRGs in UC but also strongly suggest the activation of disulfidoptosis-related cell death in UC patients.

### 2.2. WGCNA-Based Discovery of Key DRGs Involved in UC Pathogenesis and Identification of SLC3A2 as the Most Significant Central Hub

**Figure 3:**
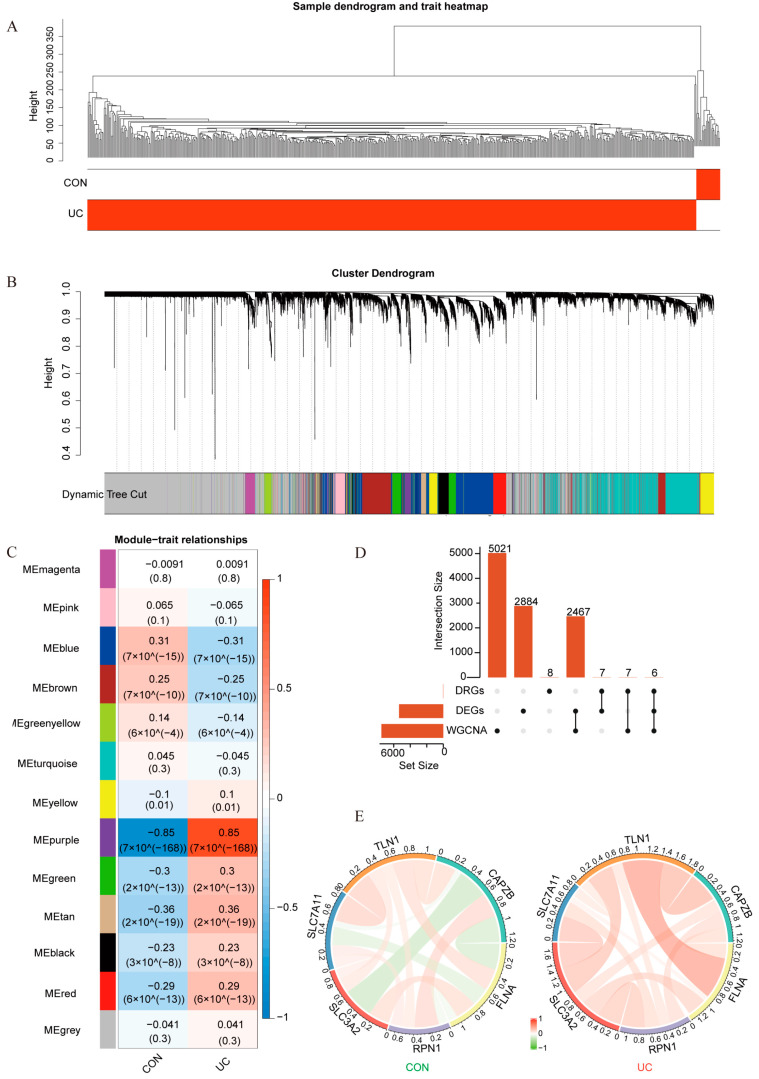
Screening for characteristic genes closely related to UC based on DRGs. (**A**) Clustering and phenotyping of samples in the dataset. Each branch in the figure represents a sample, with the vertical axis indicating the clustering distance. The closer two branches are, the more similar the corresponding samples are in terms of their gene expression profiles and clinical characteristics. The horizontal axis represents the corresponding phenotypic traits of the samples, such as disease status, clinical subtypes, or other relevant clinical features, enabling a visual assessment of how different sample groups cluster based on these traits. (**B**) Module clustering dendrogram. This graph shows different genes along the horizontal axis and the lack of correlation between genes along the vertical axis. The lower the branch, the stronger the correlation among the genes within the branch. (**C**) Heatmap illustrating the correlation between module trait genes and clinical traits. The vertical axis represents different modules, while the horizontal axis represents different traits. Each square in the heatmap represents the correlation coefficient between a specific gene module and a clinical trait, with color intensity indicating the strength of the correlation (positive or negative). The numerical values in each square represent the Pearson correlation coefficient and the corresponding *p*-value shown in parentheses. (**D**) UpSet plot showing the number of characteristic genes obtained from the intersection of the DRGs, DEGs, and the module genes identified by WGCNA. (**E**) The Circos plot illustrates the associations among characteristic genes from physiological to pathological states. Each segment represents a specific set of genes, and the connections between them indicate the relationships or correlations in gene expression. The width of the lines reflects the strength of the correlation, with color-coded links showing the direction of change (red for positive correlation, green for negative correlation).

To explore genes associated with the pathological features of UC, we utilized WGCNA. Additionally, we examined the prevalence of DRGs within the identified UC-associated gene modules, aiming to uncover potential connections between disulfidoptosis and UC pathogenesis. We clustered all samples in the training set and used the R package dplyr (1.1.4) to exclude outlier samples, which included 21 UC cases and 9 healthy controls. The results showed that the samples were well-clustered (Figure 3A).

We constructed a gene module clustering dendrogram by calculating gene similarity, determining a soft threshold of 8, and setting MEDissThres to 0.25 (Figure S3). Using the dynamic tree-cut algorithm, we identified 13 modules by merging the resulting clusters (Figure 3B). The genes positively correlated with UC, as compared to healthy controls, were selected as module genes, resulting in the identification of genes associated with disease manifestation (Figure 3C). Finally, 7501 module genes were obtained. (Table S4).

By intersecting the module genes, DEGs, and the DRGs, we identified six characteristic genes (Figure 3D; Table S5). Correlation analysis among these characteristic genes revealed that most were strongly correlated with each other. Notably, SLC3A2 (Solute Carrier Family 3 Member 2) exhibits significant differences from physiological to pathological states (Figure 3E; Table S6).

### 2.3. Machine Learning Identification of Key DRGs as Robust Diagnostic Biomarkers for UC

**Figure 4:**
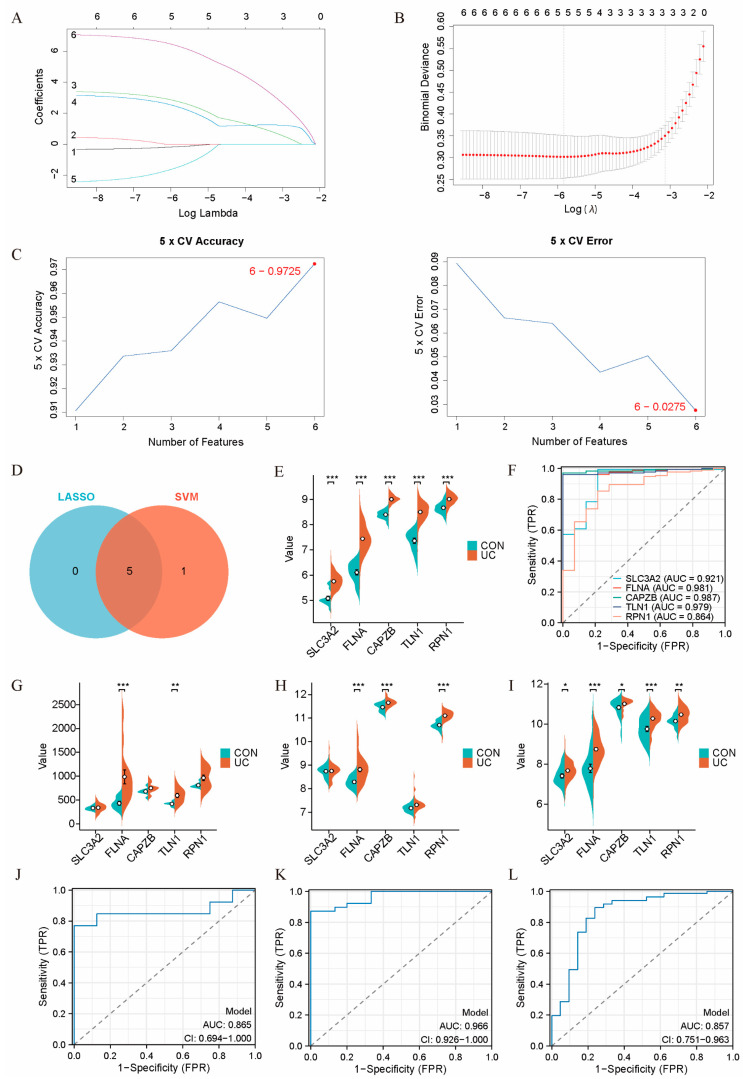
Machine learning-based screening for hub genes. (**A**,**B**) LASSO logistic regression analysis for screening hub genes. (**A**) Plot showing the changes in gene coefficients. The LASSO coefficient path plots for the six characteristic genes are displayed. Each curve represents the trajectory of a characteristic gene, with the vertical axis showing the gene value, the lower horizontal axis indicating log (λ), and the upper horizontal axis representing the number of nonzero hub genes in the model at that point. (**B**) LASSO regression cross-validation curves using a 70% training and 30% validation split to determine the optimal λ value. The two vertical lines in the plot represent key λ values. The first vertical line marks the λ value that corresponds to the point where the model begins to exhibit the least cross-validation error or where the optimal λ value is selected. The second vertical line indicates a threshold value of λ, beyond which the coefficients of some genes shrink to zero, effectively removing them from the model. (**C**) SVM analysis for screening hub genes. The plot on the left shows the accuracy of different feature combination models using fivefold cross-validation, with the horizontal axis representing the different feature combinations and the vertical axis showing the accuracy of each model. The plot on the right shows the error rate for the same models, with the horizontal axis representing the feature combinations and the vertical axis showing the error rate. Lower error rates indicate better model performance. (**D**) Venn diagram showing the intersection of hub genes identified by the two algorithms, LASSO logistic regression and SVM. (**E**,**F**) Violin plot and ROC curves revealing the expression differences and predictive power of hub genes in the 30% validation set. (**E**) The plot shows the distribution of gene expression, including the median and quartiles, providing insight into the differential expression of key genes in different conditions. (**F**) ROC curves reveal the predictive power of these hub genes in distinguishing between disease and control groups. The area under the curve (AUC) quantifies the model’s predictive accuracy, with higher AUC values indicating better predictive power. (**G**–**L**) Violin plots and ROC curves from three external datasets. (**G**) Violin plot showing expression differences in GSE48958 (CON [n = 8] vs. UC [n = 13]). (**H**) Violin plot showing expression differences in GSE47908 (CON [n = 15] vs. UC [n = 39]). (**I**) Violin plot showing expression differences in GSE92415 (CON [n = 21] vs. UC [n = 87]). (**J**) ROC curve for joint diagnostic analysis in GSE48958. (**K**) ROC curve for joint diagnostic analysis in GSE47908. (**L**) ROC curve for joint diagnostic analysis in GSE92415. * *p* < 0.05, ** *p* < 0.01, *** *p* < 0.001.

We employed LASSO and SVM algorithms to accurately identify the UC disulfidoptosis signature genes from the six characteristic genes. The application of these machine learning methods aimed to enhance the precision of feature selection, reduce model overfitting, and ensure robust identification of key genes linked to disulfidoptosis in UC. The LASSO logistic regression analysis pinpointed five hub genes: SLC3A2, FLNA, CAPZB, TLN1, and RPN1 (Figure 4A,B; Table S7). Subsequently, the SVM algorithm identified six hub genes: SLC7A11, SLC3A2, FLNA, CAPZB, TLN1, and RPN1 (Figure 4C; Supplementary Table S8). The intersection of the results from both algorithms yielded five common hub genes: SLC3A2, FLNA, CAPZB, TLN1, and RPN1 (Figure 4D).

To evaluate the diagnostic potential of these hub genes for UC, we analyzed their sensitivity and specificity using ROC curves in the training set. The results revealed that the area under the curve (AUC) values for all five hub genes were 0.9 or higher, indicating their strong diagnostic ability to differentiate between UC patients and healthy controls (Figure 4E,F; Table S9).

Further validation was conducted through ROC joint diagnostic analysis using three external datasets: GSE48958 (CON (n = 8) vs. UC (n = 13)), GSE47908 (CON (n = 15) vs. UC (n = 39)), and GSE92415 (CON (n = 21) vs. UC (n = 87)). The expression levels of the hub genes were significantly higher in the colon tissues of UC patients compared to healthy controls. The joint diagnostic analysis across different datasets demonstrated that these hub genes had robust diagnostic values, with AUC values exceeding 0.85, further confirming their potential as diagnostic biomarkers (Figure 4G–L; Table S10).

### 2.4. The Strong Correlation Between Key DRGs and Clinical Symptoms of UC, as Well as Their Response to UC Biotherapy

To investigate the correlation between disulfidoptosis-related hub genes with the varying symptoms found in UC disease, we then conducted validation studies using four external datasets, GSE47908, GSE179285, GSE75214, and GSE87466, which represent different clinical manifestations of UC patients. Additionally, we explored the potential role of these genes in predicting and modulating patient response to biologic therapies, specifically monoclonal antibodies. By examining differential gene expression in responders versus non-responders, we sought to elucidate the potential involvement of DRGs in UC pathogenesis, disease progression, and therapeutic outcomes.

**Figure 5:**
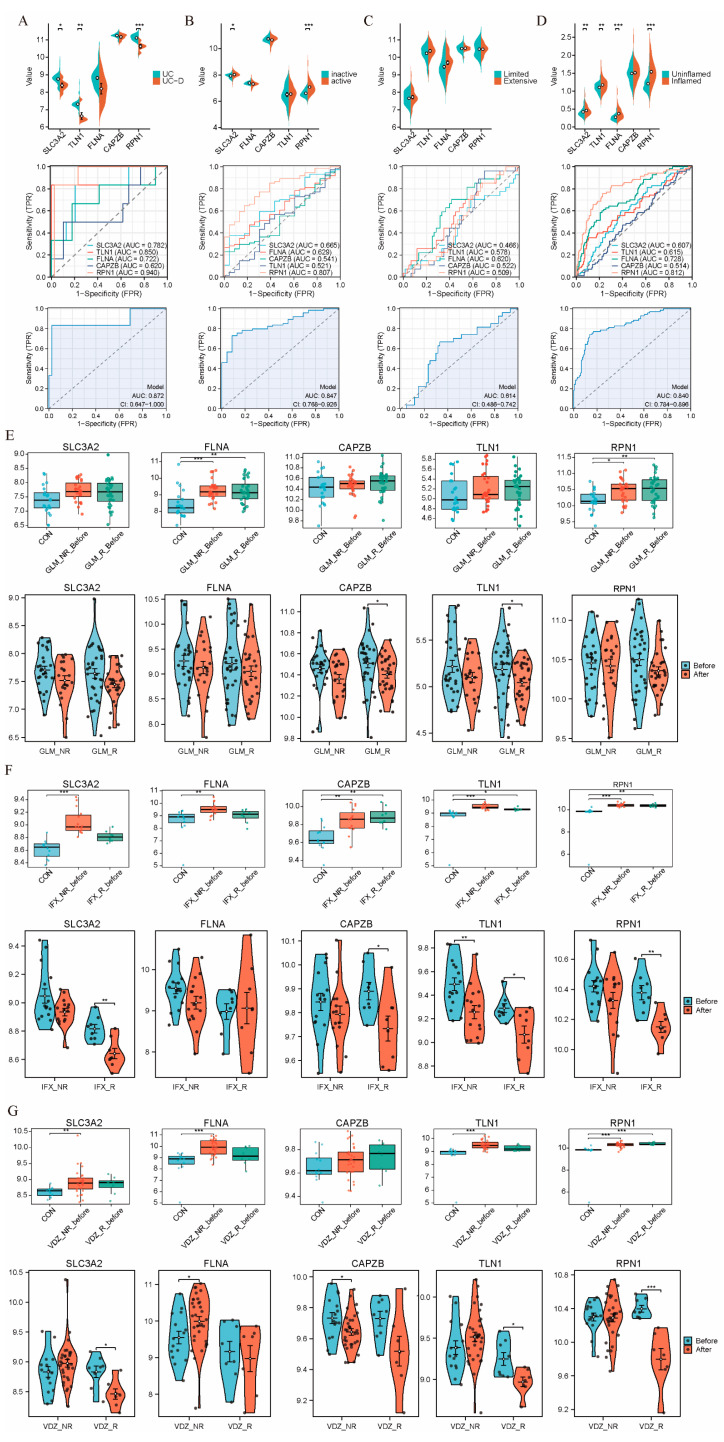
Hub genes correlate with symptoms and biologics response in UC patients. (**A**) Violin plot and ROC curves showing the expression differences and correlation of hub genes between UC (n = 39) and UC-associated dysplasia (UC-D, n = 6) in the GSE47908 validation set. The biopsy samples of UC-D were taken from areas with inflammation and dysplasia but without ulceration. (**B**) Violin plot and ROC curves showing the expression differences and correlation of hub genes between inactive UC (n = 23) and active UC (n = 74) in the GSE75214 validation set. Active disease is defined as Mayo endoscopic subscore 2 for UC patients, based on endoscopic findings. (**C**) Violin plot and ROC curves showing the expression differences and correlation of hub genes between UC with limited disease extent (n = 60) and UC with extensive disease extent (n = 27) in the GSE87466 validation set. Extensive UC refers to inflammation involving the entire colon. (**D**) Violin plot and ROC curves showing the expression differences and correlation of hub genes between non-inflammatory tissues (n = 48) and inflammatory tissues (n = 46) in the GSE179285 validation set. Biopsy samples were taken from areas of endoscopically visible mucosal inflammation, confirmed by histology. (**E**) Relative expression levels of hub genes in the colonic mucosa of healthy controls, UC patients in responding and non-responding groups before and after golimumab (GLM) therapy in the GSE92415 dataset. (**F**) Relative expression levels of hub genes in the colonic mucosa of healthy controls, UC patients in responding and non-responding groups before and after infliximab (IFX) therapy in the GSE73661 dataset. (**G**) Relative expression levels of hub genes in the colonic mucosa of healthy controls, UC patients in responding and non-responding groups before and after vedolizumab (VDZ) therapy in the GSE73661 dataset. * *p* < 0.05, ** *p* < 0.01, *** *p* < 0.001.

Firstly, the GSE47908 dataset revealed that patients with intestinal content proliferation exhibited downregulation of all five characteristic hub genes. ROC analysis showed that the multigene combination of these hubs achieved an AUC of 0.872, highlighting a significant association between these genes and intestinal content proliferation, with SLC3A2, TLN1, and RPN1 showing notable performance in single-gene ROC analysis (Figure 5A). In the GSE75214 dataset, which differentiates active and inactive disease phases, significant differences in key gene expression were observed, with an AUC of 0.847, further supporting the correlation between these hub genes and UC’s clinical manifestations. SLC3A2 and RPN1 once again showed significant results in the single-gene ROC analysis (Figure 5B). For the GSE179285 dataset, our analysis demonstrated a marked increase in hub gene expression under inflammatory conditions, with an AUC of 0.840, underscoring a strong association between these key genes and intestinal inflammation. RPN1 stood out in the single-gene ROC analysis (Figure 5D). In contrast, the GSE87466 dataset showed no significant differences in hub gene expression between localized and diffuse ulcerative lesions (Figure 5C). DRGs show a significant upregulation in the inflammatory and acute phases of UC, but an abnormal downregulation in expression associated with the presence of intestinal content proliferation, compared to the pathological state. This suggests that disulfidoptosis plays a crucial role in the acute and inflammatory phases of ulcerative colitis, with its expression being altered during intestinal proliferation.

To explore the response of DRGs to biologic therapies, such as TNF-α inhibitors golimumab (GLM), infliximab (IFX), and vedolizumab (VDZ), we analyzed the GSE92415 and GSE73661 datasets, which include colon biopsy samples from patients with moderate to severe colitis. In the GSE92415 dataset, which includes UC patients treated with GLM, hub gene expression was elevated before treatment (Figure 5E). Following GLM therapy, CAPZB and TLN1 were significantly downregulated, with RPN1 also showing a downward trend. Similarly, the GSE73661 dataset, comprising UC patients treated with IFX, revealed higher pre-treatment expression of hub genes compared to healthy controls. (Figure 5F). Post-IFX treatment, SLC3A2, CAPZB, TLN1, and RPN1 showed notable downregulation. The same dataset for VDZ treatment also demonstrated significant downregulation of SLC3A2, TLN1, and RPN1 after therapy (Figure 5G). Interestingly, patients who did not respond to these treatments generally had higher baseline levels of DRGs. These findings suggest that DRGs are associated with the severity of the disease and in patients who respond to biologic therapies, including GLM, IFX, and VDZ; this treatment may alleviate colonic damage in UC patients by modulating disulfidoptosis-related pathways.

### 2.5. Significant Correlation of Disulfidoptosis-Related Hub Genes with Immune Cell Infiltration in UC

**Figure 6:**
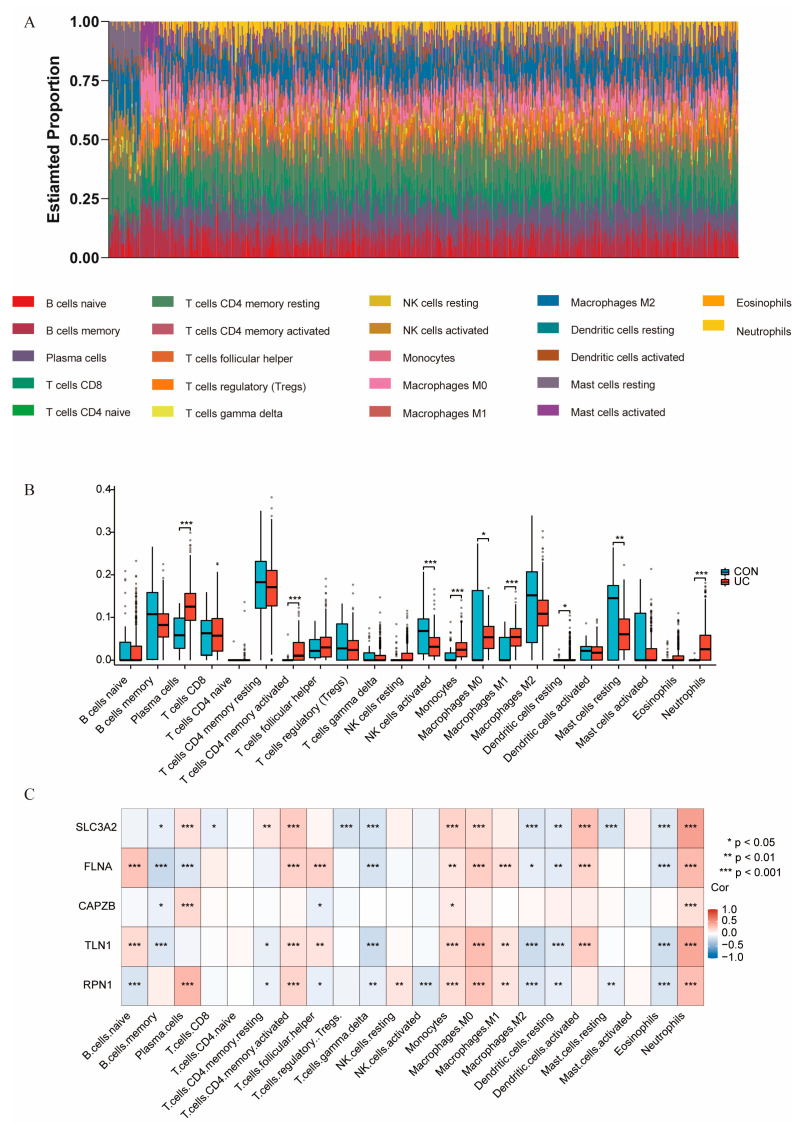
Estimation of infiltrating immune cell types and correlation analysis with hub genes. (**A**) Bar plots showing the relative composition of 22 immune cell subsets in the training sets. Each bar represents the proportion of a specific immune cell subset across the samples in the training set. The immune cell types are categorized based on immune classification, and the height of each bar corresponds to the relative abundance of each subset within the sample population. (**B**) Box plots illustrate the differences in the proportion of immune cells between the control (CON) and UC groups. Each box plot displays the distribution of immune cell proportions within each group, with the boxes representing the IQR and the whiskers showing the range of values. (**C**) Correlation analysis of hub genes with the 22 immune cell types. Data were assessed using the Benjamini and Hochberg (BH) method. * *p* < 0.05, ** *p* < 0.01, *** *p* < 0.001.

Previous findings have shown that disulfidoptosis signaling is significantly activated in inflammatory colonic tissue. To further investigate this, we analyzed the immune landscape of the training sets using CIBERSORTx, visualizing the abundance of 22 immune cell types with bar plots. As illustrated in the figure, the main immune cell composition of the colon consists of plasma cells, B cells, mast cells, T cells, macrophages, and neutrophils, along with a small proportion of NK cells and dendritic cells (Figure 6A).

Our analysis revealed significant differences in nine immune cell types between UC samples and control samples (*p* < 0.05) (Table S11). Specifically, plasma cells, activated CD4 memory T cells, monocytes, M1 macrophages, resting dendritic cells, and neutrophils were significantly more prevalent in UC tissues. In contrast, activated NK cells, M0 macrophages, and resting mast cells were found to be significantly less abundant in UC patients compared to healthy controls (Figure 6B).

**Figure 7:**
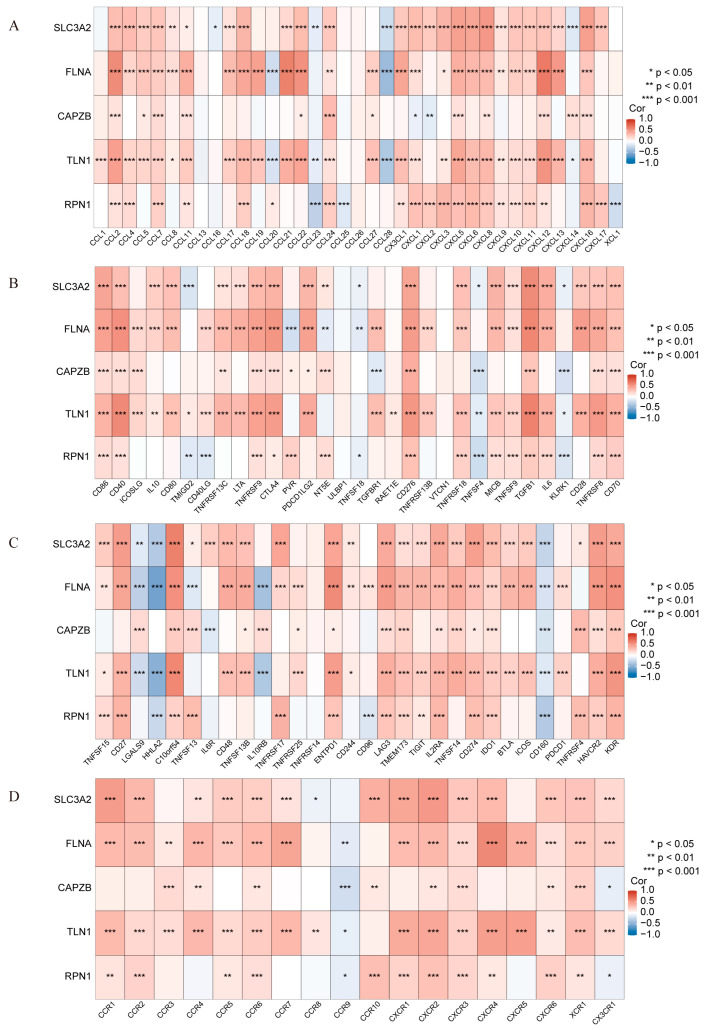
Correlation Analysis of Hub Genes and Immunostimulatory Factors. (**A**) Correlation analysis of hub genes with chemokines. (**B**,**C**) Correlation analysis of hub genes with immunoinhibitors. (**D**) Correlation analysis of hub genes with receptors. (**D**) The protein expression levels of Slc3a2 (n = 3), * *p* < 0.05, ** *p* < 0.01, *** *p* < 0.001.

We further conducted a correlation analysis on the expression data from the training set, identifying that hub genes were positively correlated with most pro-inflammatory factors (Figure 7A, Table S12), immunoinhibitors (Figure 7B,C, Table S13), and receptors (Figure 7D, Table S14). Additionally, the relationship between hub genes and immune cells was explored, revealing that most hub genes positively correlated with pro-inflammatory cells, including neutrophils, M1 macrophages, monocytes, activated CD4 memory T cells, and activated dendritic cells, while showing negative correlations with anti-inflammatory cells such as M2 macrophages (Figure 6C). The strong correlations indicate that DRGs play pivotal roles in modulating immune responses, maintaining immune balance, and influencing inflammatory processes.

These analyses highlight the changes in immune cells in UC and underscore the close relationship between disulfidoptosis-related hub genes, immune cells, and immune regulatory factors, emphasizing the need for further investigation into their mechanisms of action.

### 2.6. Construction of a CeRNA Network Reveals Regulatory Mechanisms of Key Disulfidoptosis-Related Hub Genes in UC

Having identified key hub genes with disulfide bond formation and confirmed their critical roles in disease progression, drug response, and immune-related mechanisms, we next sought to understand the regulatory pathways governing these hub genes. To this end, we conducted a comprehensive RNA analysis to further elucidate their underlying regulatory mechanisms. The competing endogenous RNA (ceRNA) hypothesis [12] proposes a regulatory mechanism in which microRNAs (miRNAs) not only regulate messenger RNA (mRNA) but also compete with long non-coding RNAs (lncRNAs) for binding, thereby influencing mRNA expression levels [13]. To elucidate the molecular mechanisms underlying disulfidptosis in UC patients, we aimed to construct a ceRNA network based on significant changes in hub genes.

**Figure 8:**
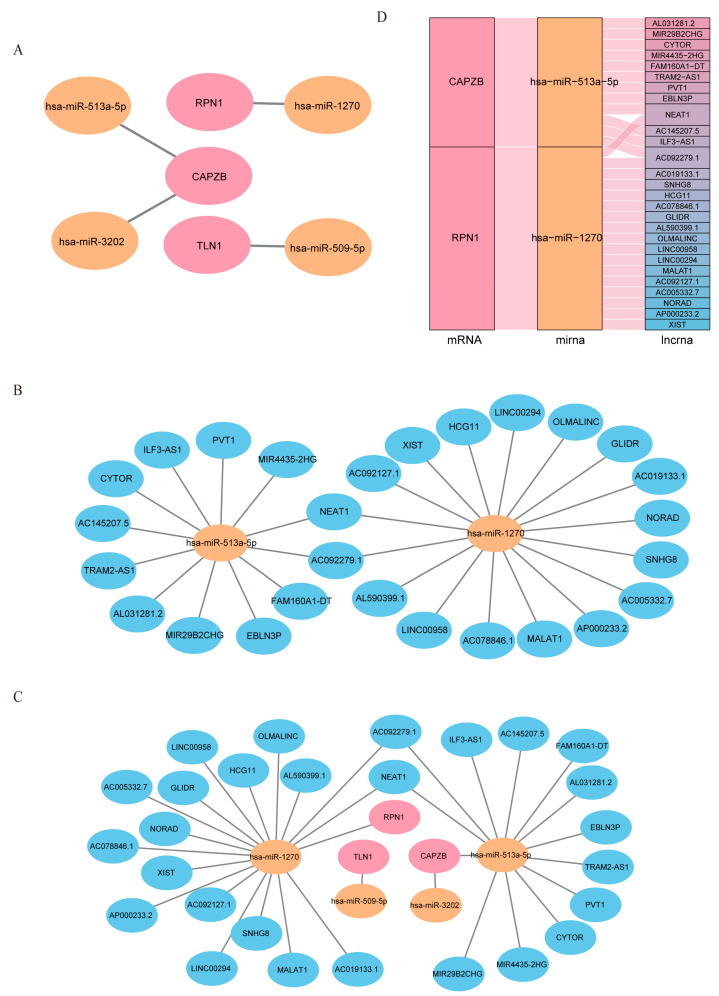
Reliable CeRNA Network Construction. (**A**) miRNA–mRNA network including 3 hub genes and 3 regulatory miRNAs. (**B**) Prediction of 27 experimentally verified (AGO-CLIP > 1 and degradome-seq > 1) lncRNAs from 2 out of the 3 validated miRNAs using the ENCORI database. (**C**) Construction of the reliable lncRNA–miRNA–mRNA network, featuring 3 hub genes, 4 miRNAs, and 27 lncRNAs. Light orange nodes represent hub genes, light pink nodes represent miRNAs, and light blue nodes represent lncRNAs. (**D**) Sankey diagram of the final CeRNA network, where the squareness represents lncRNAs, miRNAs, and mRNAs, and the size indicates their degree of connection.

Using multiMiR prediction, we identified 373 experimentally validated miRNAs targeting the hub genes (Table S15). After analyzing their expression in the GSE48957 dataset, we focused on downregulated miRNAs (logFC < −0.05) to construct a reliable hub gene–miRNA network (Figure 8A). We then predicted 27 experimentally verified lncRNAs interacting with these miRNAs using the ENCORI database. Following expression screening in the GSE77013 dataset, we retained two of four detectable miRNAs in colon tissues (Figure 8B).

Ultimately, we constructed a credible ceRNA network comprising 3 mRNAs, 4 miRNAs, and 27 lncRNAs (Figure 8C), providing valuable insights into the regulatory mechanisms of disulfidptosis in UC (Figure 8D).

### 2.7. Adrenergic Receptor Compound Identification and Molecular Docking Studies Highlight Clonidine’s Interaction with SLC3A2 in UC Management

**Figure 9:**
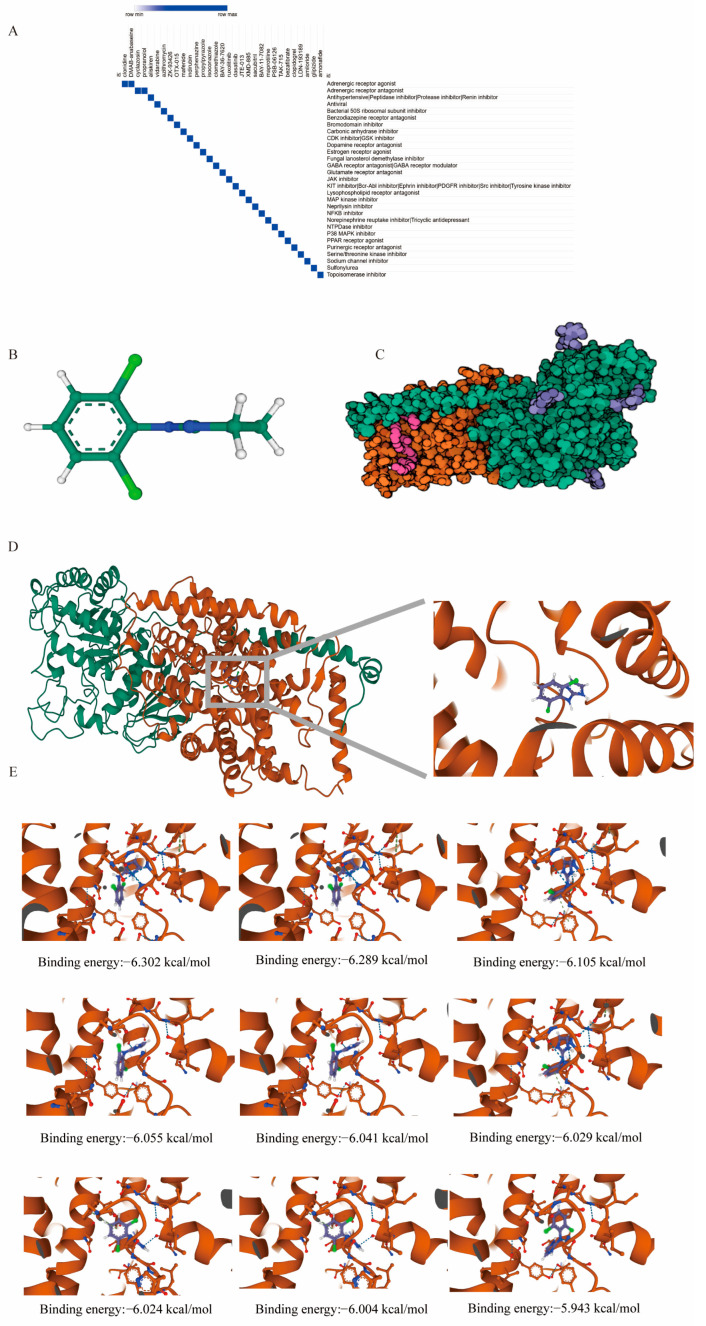
Prediction of potential drugs targeting disulfidptosis signaling. (**A**) Identification of potential small molecule compounds for managing UC using the Connectivity Map (CMap) database. The top 31 small molecules (normalized connectivity score, ncs < −1.8) were identified by submitting the up-regulated DRGs to CMap. A false discovery rate (FDR) cutoff of q_nlog10 > 15 was applied to filter the compounds that exhibited reverse perturbations to the hub genes. (**B**) Three-dimensional structure of clonidine from the PubChem database. (**C**) Three-dimensional structure of SLC3A2 (PDB ID: 7CMH) with high-resolution data (3.4 Å) obtained from the RCSB Protein Data Bank. (**D**) Docking model of the Clonidine-SLC3A2 protein–ligand complex. The protein (SLC3A2) and ligand (Clonidine) files were converted to PDBQT format, excluding water molecules and adding polar hydrogens. The docking grid box was set to 30 Å × 30 Å × 30 Å with a 0.05 nm grid point spacing centered on the protein’s domain to allow free movement of the ligand. All docking studies were performed using AutoDock Vina 1.2.2 to calculate binding affinities and predict the most likely binding conformations of Clonidine with the SLC3A2 protein. (**E**) Binding energy analysis between Clonidine and SLC3A2. The top 9 binding poses with the lowest binding energies are shown, calculated using AutoDock Vina 1.2.2. The binding energies (in kcal/mol) for each pose are indicated, with more negative values representing stronger binding interactions.

We submitted the up-regulated DRGs to the Connectivity Map (CMap) database to identify potential small molecule compounds for managing UC. Using a false discovery rate (FDR) cutoff of q_nlog10 > 15, we identified the top 31 small molecules (normalized connectivity score, ncs < −1.8) that exhibited reverse perturbations to the hub genes (Table S16). Adrenergic receptor agonists and antagonists were the most relevant compounds (Figure 9A). The compound with the lowest ncs was further analyzed, and its structure was retrieved from the PubChem database (Figure 9B). To investigate its potential interaction with the key hub gene SLC3A2 (Figure 9C), molecular docking studies were performed (Figure 9D). Molecular docking, a key technique for structure-based drug design, aims to identify the optimal conformation of small molecules bound to their target proteins.

For this study, we used high-resolution structural data (3.4 Å) for SLC3A2 (PDB ID: 7CMH) from the RCSB Protein Data Bank and conducted docking with clonidine. The binding energy between the clonidine and SLC3A2 was calculated to be below −5 kcal/mol (Figure 9E), indicating a relatively strong interaction. This suggests that the drug may bind to the protein with significant affinity, potentially resulting in stable complex formation.

### 2.8. DSS-Induced Mouse Colitis Reveals Upregulation of Key Hub Genes, Supporting the SLC3A2 as a Disulfidoptosis-Related Signaling in UC

**Figure 10:**
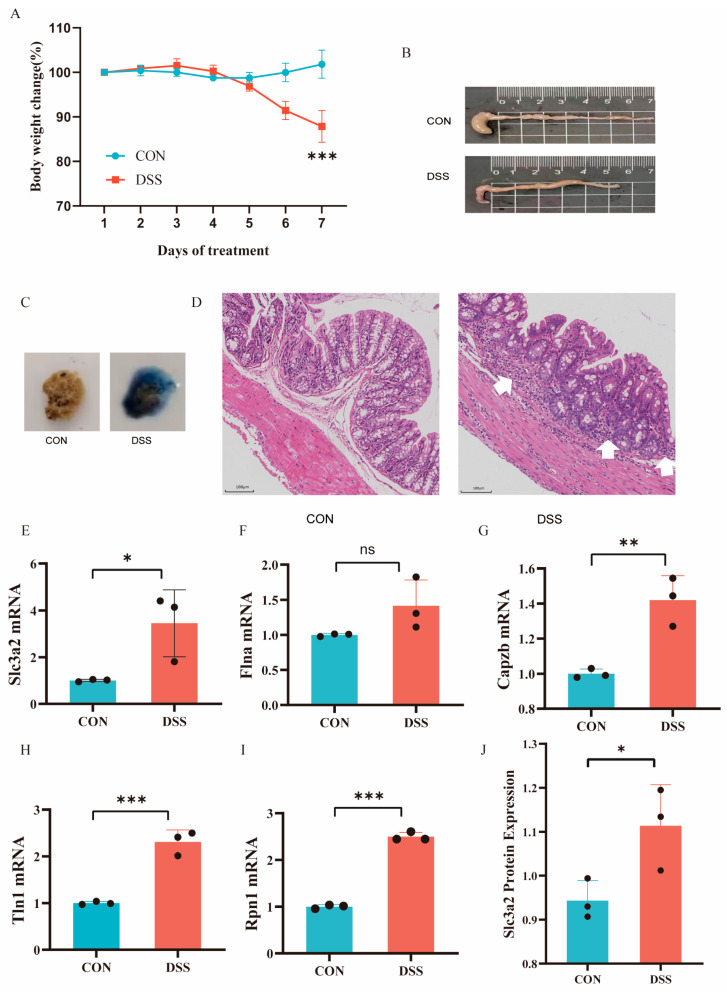
Animal models validate the core genes of disulfidptosis signaling. (**A**) Body weight changes (n = 4–5). (**B**) Colonic length changes (n = 4–5) between CON and UC mice. (**C**) Typical fecal occult blood test (FOBT) results in CON and DSS groups. (**D**) Representative images of HE staining in the colon tissues (magnification ×100) and arrows represents inflammation. (**E**–**I**) The mRNA expression levels of Slc3a2 (**E**), Tln1 (**F**), Capzb (**G**), Flna (**H**), and RPN1 (**I**) in UC and CON samples by RT-qPCR (n = 3). (**J**) The protein expression level of Slc3a2. Data are shown as mean ± SD. ns, not significant, * p < 0.05, ** p < 0.01, *** p < 0.001.

To further validate the important role of hub genes in UC, we employed the DSS-induced mouse colitis model. Compared with the CON group, mice in the DSS group showed obvious weight loss (Figure 10A), decreased colon length (Figure 10B), and bloody stools (Figure 10C). The histopathological examination showed severe inflammatory infiltrates and disruption of the intestinal mucosa in the DSS group (Figure 10D). Then, we detected the expression levels of hub genes using RT-qPCR. Compared with the normal group, the expression levels of Slc3a2, Tln1, Capzb, Flna, and Rpn1 in the UC group were significantly increased, with Flna showing an upward trend (Figure 10E–I). The proteomics study found that SLC3A2 is upregulated in the intestines of UC patients (Figure 10J), and the consistency between protein and RNA changes indicates that the key disulfidoptosis gene SLC3A2 plays an important role in UC. These results provide cross-species evidence supporting the function and regulation of disulfidptosis signaling in UC.

## 3. Discussion

Ulcerative colitis is a complex disease characterized by intricate pathogenesis and limited treatment efficacy; patients often experience malnutrition in the later stages of the disease. Disulfidptosis, a newly discovered form of cell death, is characterized by the abnormal accumulation of disulfide bonds in cytoskeletal proteins and dysregulated oxidative stress under glucose deprivation, ultimately leading to cell death. Studies have shown that cytoskeletal proteins in the intestine play a crucial role in coordinating endocytosis and exocytosis, thereby contributing significantly to nutrient absorption and intercellular material exchange [14,15].

In this study, we integrated large-scale datasets, removing batch effects and outliers to investigate the general expression patterns of DRGs in UC. This approach enhanced the reliability of our data. The integration results showed that our large dataset aligned with common UC characteristics, with significant differences in the expression of DRGs between UC patients and healthy controls. Currently, 28 DRGs have been reported, including 17 actin-associated genes, 4 inhibitory factors, glycogen synthase 1 (GYS1), and several genes involved in mitochondrial oxidative phosphorylation (e.g., NDUFS1, NDUFA11, NUBPL, and LRPPRC), as well as MYH9 and MYH10. Among these, 21 DRGs were found to be differentially expressed in UC, encompassing all inhibitory factors, DRGs related to oxidative phosphorylation and glucose metabolism, and more than half of the 17 actin-associated genes.

Genes such as GYS1 have been shown to play a role in cellular survival during glucose deprivation by promoting a shift toward oxidative phosphorylation and glycogen metabolism [16]. This phenomenon aligns with the observed increase in oxidative phosphorylation and enhanced oxidative stress responses seen in UC. The cytoskeleton plays various roles in the intestine, such as providing structural support to cells by linking microtubules and microfilaments, thereby maintaining the shape and integrity of intestinal epithelial cells and facilitating cell migration and motility, especially during intestinal repair [17,18,19]. Moreover, cytoskeletal proteins interact with signal transduction pathways to regulate cellular physiological responses and metabolic activities [20,21]. Filamin A (FLNA), a cytoskeletal protein connecting the cell membrane and cytoskeleton, plays a critical role in cell migration, shape maintenance, and signal transduction, influencing inflammatory diseases through its regulation of cell adhesion and migration [22,23,24]. Ge Gen Qin Lian Decoction (GGQLT) has been found to have a clear effect in alleviating intestinal inflammation in UC, and this effect is specifically mediated through the modulation of Talin 1 (TLN1) [25]; recent findings suggest that puerarin may also act on intestinal villi to enhance metabolic capacity in the gut [26]. These findings indicate a potential link between DRGs and intestinal absorption and metabolism.

Using WGCNA, we identified gene modules in UC that were associated with disease phenotypes compared to healthy individuals. By intersecting the module genes, DEGs, and the DRGs, we identified six characteristic genes. These six genes are DRGs that exhibit significant expression differences in UC and are related to UC disease phenotypes. Further validation using machine learning refined these findings, identifying five hub genes. We validated these five genes across three external datasets of varying sample sizes and found that they had strong predictive and diagnostic value for UC. At the same time, we validated the expression of the hub genes in four clinical symptom-related datasets and two biologic-related datasets. The results suggest that disulfidoptosis plays a crucial role in the acute and inflammatory phases of UC. These analyses demonstrate that disulfidptosis is significantly expressed in UC and is strongly associated with disease characteristics; the more pronounced the inflammation, the more prominently DRGs are expressed in UC.

Through immune infiltration analysis and the correlation analysis between DRGs and immune cells, we found that the key DRGs have a strong correlation with both innate and adaptive immune responses. Notably, there is a significant association with monocytes, such as macrophages and neutrophils. Additionally, studies have reported aberrant expression of cytoskeletal protein-associated factors in UC [20], which are closely linked to immune cell interactions [27,28,29,30,31]. TLN1, as an integrin-activating protein, mediates interactions between cells and the extracellular matrix (ECM). The components of the ECM can induce macrophage differentiation into different phenotypes, and abnormal TLN1 expression may lead to ECM dysfunction and promote inflammatory macrophage differentiation [32]. Therefore, investigating the role of disulfidptosis in UC may provide crucial insights into the disease’s underlying mechanisms.

We also analyzed the correlation between the expression of DRGs and UC patients’ responses to TNF-α inhibitors. The results revealed that patients who did not respond to TNF-α inhibitors exhibited higher expression levels of DRGs in the early stages of treatment (which may have caused irreversible damage to the intestine’s self-repair functions). In contrast, patients who responded to the treatment showed partial downregulation of DRGs pre- and post-treatment. However, current biologics do not specifically target the regulation of DRGs, indicating the necessity to develop drugs targeting disulfidptosis to alleviate inflammation and slow disease progression in UC.

The current study also successfully screened SLC3A2 as a potential small molecular target, which should be investigated in the future. Firstly, we found that SLC3A2 showed a strong positive correlation with plasma cells, memory T cells, monocytes, M0 macrophages, activated dendritic cells, and neutrophils while displaying a negative correlation with regulatory T cells, M2 macrophages, resting dendritic cells, mast cells, and eosinophils. Next, through small molecule drug screening, we found that the upregulation of DRGs could be suppressed by clonidine, which reduces sympathetic activity.

Interestingly, the following study demonstrated a relatively strong interaction between SLC3A2 and clonidine, indicating that SLC3A2 may be a potential small molecule that regulates or influences sympathetic activity. Previous studies have shown that UC patients frequently exhibit autonomic nervous dysfunction [33], characterized by increased sympathetic tone and decreased norepinephrine release [34]. In UC models, the sympathetic nervous system, primarily via the splenic nerve pathway, is involved in the inflammatory immune response. Both vagotomy and splenic nerve transection have been shown to exacerbate inflammation [35]. Last but not least, higher Slc3a2 expression was found in the DSS mice colon. Given that SLC3A2 is a member of the solute carrier family, it has been implicated in tumorigenesis, metabolic diseases, and inflammatory responses. Additionally, SLC3A2 encodes the molecular chaperone of SLC7A11 and high SLC7A11 expression, potentially leading to F-actin contraction and detachment from the plasma membrane [36,37]. Therefore, our findings highlight the importance of exploring the mechanisms by which disulfidptosis contributes to UC pathology and suggest that targeting sympathetic activity by SLC3A2 may be a potential pathway in treating UC.

Our study primarily relied on big data mining; however, the lack of detailed demographic information (such as age, sex, and disease duration) in the datasets limited the depth of our analysis. For instance, we were unable to investigate whether there are gender differences or age-related susceptibility to disulfidoptosis in UC, or whether disulfidoptosis varies at different stages of disease progression. This limitation may hinder the ability to conduct a more detailed analysis of the pathophysiology of the disease. Additionally, in the drug response analysis, we focused on studying the expression of disulfidoptosis-related genes in patients with moderate to severe UC. Although this approach is consistent with our hypothesis that more severe disease may cause irreversible damage, thereby affecting the response to biologics, the lack of comprehensive demographic data in the dataset hinders further exploration of these relationships. Transcriptome research in the future should include more comprehensive demographic data to facilitate more thorough analysis. Moreover, further research is needed on the interaction between SLC3A2 and the sympathetic nervous system in order to deepen our understanding of how SLC3A2 is involved in the pathogenesis of UC and potentially reveal new therapeutic targets for regulating immune responses.

## 4. Materials and Methods

### 4.1. Datasets and Sample Selection

We searched GEO databases using the keyword “ulcerative colitis”, and the filter criteria were as follows: ① human; ② UC patients with an established diagnosis; and ③ the dataset had at least five healthy control and five UC samples. Finally, seven mRNA datasets (GSE193677, GSE206285, GSE87466, GSE66407, GSE128682, GSE73661, GSE92415), one miRNA dataset (GSE48957), and one lncRNA dataset (GSE77013) were included (Table 1) [38,39,40,41,42,43,44,45].

The GSE179285 dataset contained colon tissue biopsy samples, which included 31 healthy control samples and 23 UC samples, and the GSE206285 dataset contained colon tissue biopsy samples from 18 healthy individuals and 550 UC patients. The GSE179285 dataset was annotated by the GPL6480 Agilent-014850 Whole Human Genome Microarray 4 × 44K G4112F (Probe Name version) platform, while the GSE206286 dataset was annotated by the GPL13158[HT_HG-U133_Plus_PM] Affymetrix HT HG-U133+ PM Array Plate. merged by batch-effects processing and used as a training set, which contained 49 healthy control samples and 573 UC samples for subsequent analysis (Figure S1). Relevant details of the validation sets are summarized in Tables S1 and S2, Figure S2.

DRGs were obtained according to the published literature (PMID: 36747082), specifically SLC7A11, SLC3A2, NCKAP1, WASF2, CYFIP1, ABI2, BRK1, NUBPL, NDUFA11, LRPPRC, OXSM, NDUFS1, GYS1, FLNA, FLNB, MYL6, MYH9, MYH10, ACTB, ACTN4, CAPZB, CD2AP, DSTN, TLN1, INF2, PDLIM1, IQGAP1, and RPN1 [4].

### 4.2. Identification of Differentially Expressed Genes (DEGs)

The Limma (3.58.1) R package was used to obtain the genes expressed differently between UC samples and healthy control samples from microarray datasets. The adjusted *p*-value < 0.05 and a |log2 (fold change)| ≥ log2 (1.2) were considered to be statistically significant [46,47,48]. Volcano plots of the results were drawn through the R package ggplot (3.5.1). Box line plots of the results were drawn through xiantaozi (https://www.xiantaozi.com/, accessed on 20 August 2024), a comprehensive web service for biomedical data analysis and visualization.

### 4.3. Biological Function and Pathway Enrichment Analysis

Gene Ontology (GO) analysis and Kyoto Encyclopedia of Genes and Genomes (KEGG) pathway enrichment analysis were performed using the xiantaozi and visualization by weishengxin (http://www.bioinformatics.com.cn/, accessed on 20 August 2024).

### 4.4. Construction of the Coexpression Network and Screening of the Module Genes

To find modular genes in the training set that are highly correlated with the phenotype, WGCNA was performed on all genes using the UC and healthy controls as phenotypes. The samples were first clustered, and outliers were removed. The soft threshold for the data was determined (power = 8, −R^2^ = 0.85) to ensure that gene interactions maximally conformed to a scale-free distribution (Figure S3A,B). Then, we constructed a coexpression matrix, calculated the proximity and similarity between genes to construct a systematic clustering tree of genes, and identified gene modules by hierarchical clustering. MEDissThres was set to 0.25 to merge similar modules analyzed by the dynamic shear tree algorithm, and the relevant modules were screened with UC and healthy controls as key modules (Figure S3C). The module genes were obtained by combining the genes of the key modules.

### 4.5. Screening of Characteristic Genes and Their Correlation Analysis

The intersection of DEGs, module genes, and DRGs was taken to obtain characteristic genes using the R package UpSetR (1.4.0). The correlation between the intersected genes was calculated in the training set by the Pearson algorithm to obtain the corresponding *p*-value and correlation coefficients.

### 4.6. Screening of Hub Genes and Evaluation of Diagnostic Performance

The LASSO algorithm and SVM algorithm were used to identify the candidate genes in the training set. The R package Venn Diagram (1.7.3) was used to intersect the genes identified by the two algorithms to obtain the hub genes, and then the R package p ROC (0.7.0) was used to analyze the ROC curves of the genes and to verify their diagnostic value.

### 4.7. Evaluation of Tissue Infiltrating Immune Cells

Immune cell infiltration analysis was carried out using the Cibersort (https://cibersortx.stanford.edu/, accessed on 21 August 2024), which can predict the immune cell composition of tissues, deconvolution algorithm based on input gene expression profiles, and the builtin reference set LM22. Permutation (PERM) was established to 100 for more stable results [49,50,51].

### 4.8. Correction Analysis

The Hmisc (5.1-0) package was used to complete the correlation analysis between genes and the correlation analysis between genes and immune cells, genes, and immunostimulatory factors. The immunostimulatory factors were downloaded from the organized gene list of “Immunomodulator” of TISIDB (http://cis.hku.hk/TISIDB/index.php, accessed on 21 August 2024). Additionally, all correlation heatmap visualizations are performed with xiantaozi.

### 4.9. CeRNA Network Construction

The multiMiR (1.20.0) package was used to predict interactions between hub genes and miRNAs, which compiled nearly 50 million records in humans and mice from 14 databases [52]. Choose all miRNAs experimentally validated from the list of predicted records. ENCORI (https://rnasysu.com/encori/, accessed on 21 August 2024) was used to predict miRNA–lncRNA interactions with the screening conditions AGO-CLIP and degradome-seq both at >1. The interaction networks were constructed and visualized using Cytoscape, and weishengxin drew a Sankey diagram.

### 4.10. Small Molecule Agent Screening and Molecular Docking Analysis

The Connectivity Map database (CMap, https://clue.io/, accessed on 31 August 2024) is a drug prediction database based on differential gene expression, primarily used to explore functional relationships among genes, small molecule compounds, and diseases [53,54,55,56].

Home for Researchers (www.home-for-researchers.com, accessed on 31 August 2024) was used to perform molecular docking of the key targets with small molecule compounds. To analyze the binding affinities and modes of interaction between the drug candidates and their targets, Autodock Vina 1.2.2, a computational protein–ligand docking software, was employed [57]. The molecular structure of clonidine was retrieved from PubChem Compound (https:/pubchem.ncbi.nlm.nih.gov/, accessed on 30 August 2024) [58]. The 3D coordinates of SLC3A2 (PDB ID: 7CMH; resolution: 3.4 Å) were downloaded from The Protein Data Bank (http://www.rcsb.org, PDB, accessed on 31 August 2024). For the docking analysis, all protein and molecular files were converted to the PDBQT format, with all water molecules excluded and polar hydrogen atoms added. The grid box was centered to encompass the protein’s domain and to allow free movement of the molecules. The grid box was set to 30 Å × 30 Å × 30 Å, with a grid point spacing of 0.05 nm. Molecular docking studies were conducted using Autodock Vina 1.2.2 (http://autodock.scripps.edu/, accessed on 30 August 2024).

### 4.11. Animal Model of Colitis

Male C57BL/6J mice (~25 g) were obtained from Gempharmatech (Chengdu, China).

This study was performed in accordance with the recommendations in the Guide for the Care and Use of Laboratory Animals of the National Institutes of Health. The protocol was approved by the Committee on the Ethics of Animal Experiments of Chengdu University of Traditional Chinese Medicine. Mice were randomized into the control group and DSS group. Mice in the DSS group were provided with 2.5% DSS (MP Biomedicals, Shanghai, China) in their drinking water for 7 days [59], and the control group was only administered distilled water. Body weight was measured daily, and fecal occult blood was assessed on day 7.

### 4.12. H&E Staining

Entire colons were excised postmortem. Colon tissues were fixed with 4% paraformaldehyde (PFA) overnight and were then embedded in paraffin. Colonic sections of 5 mm were obtained and laid flat on a glass slide for H&E staining. The results were imaged by Pannoramic 250FLASH (3DHISTECH, Budapest, Hungary).

### 4.13. RNA Extraction and Quantitative Real-Time PCR (qRT-PCR)

RNA was extracted from the intestinal tissues using the Animal Total RNA Isolation Kit (Foregene Biotechnology, Co., Ltd., Chengdu, China) and reverse-transcribed to cDNA using the RT Easy TM II (Foregene Biotechnology, Co., Ltd., Chengdu, Sichuan, China). Primer sequences were shown in Table 2 and synthesized at Tsingke (Beijing Tsingke Biotech Co., Ltd., Beijing, China). RT-PCR was performed using the Real-Time PCR Easy TM-SYBR Green I (Foregene Biotechnology, Co., Ltd., Chengdu, China) on a QuantGene 9600 (Bioer Technology, Hangzhou, China). PCR amplification was conducted in triplicate for each sample, and the expression of target genes was normalized to β-actin. Relative expression was determined using the 2^−∆∆Ct^ method.

### 4.14. Protein Extraction and Mass Spectrometry Analysis

Samples were ground into cell powder using liquid nitrogen and lysed in a buffer containing urea (Sigma-Aldrich, St. Louis, MO, USA), protease inhibitors (Calbiochem, San Diego, CA, USA), TSA (Sigma-Aldrich, St. Louis, MO, USA), and NAM (Sigma-Aldrich, St. Louis, MO, USA). After sonication and centrifugation, the supernatant was collected, and protein concentration was determined using a BCA kit (Beyotime Biotechnology, Co., Ltd., Shanghai, China). Proteins were reduced with dithiothreitol (Sigma-Aldrich, St. Louis, MO, USA), alkylated with iodoacetamide (Sigma-Aldrich, St. Louis, MO, USA), and digested with trypsin (Sigma-Aldrich, St. Louis, MO, USA). Peptides were desalted, vacuum-dried, and labeled with a TMT kit (Thermo Fisher Scientific, Waltham, MA, USA) according to the manufacturer’s protocol. The labeled peptides were separated on a reversed-phase column using an EASY-nLC 1000 UPLC system and analyzed by tandem mass spectrometry (MS/MS) on a Q Exactive Plus instrument. MS/MS data were acquired using a data-dependent method, and peptides were identified and quantified in the Orbitrap at high resolution.

### 4.15. Statistical Analysis

All data analyses were conducted with GraphPad Prism V8.0 software. Data are presented as mean ± SD. Student’s *t*-tests were applied for comparisons between two groups, and repeatedly measured data were analyzed by repeated measurement analysis of variance. All statistical analyses were two-sided, and *p* < 0.05 was considered to be significant.

## 5. Conclusions

Our study identified key genes that establish a link between disulfidptosis and UC, and we constructed a corresponding ceRNA network for further analysis. We also investigated the association between these key genes and potential therapeutic agents. Disulfidptosis is highly expressed during the inflammatory phase of UC and is closely linked to patient responses to biologic therapies. It is valuable for understanding UC inflammation, disease progression, and prognosis. Overall, our study underscores the pivotal role of disulfidptosis in UC pathogenesis, with the expression of related genes serving as potential targets for disease prediction and therapy. Notably, SLC3A2 emerges as a promising candidate for future investigation.

## Figures and Tables

**Table 1:** The information of all the datasets in the study.

GEO ID	RNA Type	Platform	Tissues	Attribute
GSE179285	mRNA	GPL6480	Colonic mucosal	Training set
GSE206285	mRNA	GPL13158	Colonic mucosal	Training set
GSE48958	mRNA	GPL6244	Colonic mucosal	Validation set
GSE92415	mRNA	GPL13158	Colonic mucosal	Validation set
GSE47908	mRNA	GPL570	Colonic mucosal	Validation set
GSE75214	mRNA	GPL6244	Colonic mucosal	Validation set
GSE87466	mRNA	GPL13158	Colonic mucosal	Validation set
GSE73661	mRNA	GPL6244	Colonic mucosal	Validation set
GSE48957	miRNA	GPL14613	Colonic mucosal	Validation set
GSE77013	lncRNA	GPL16956	Colonic mucosal	Validation set

**Table 2:** The primer sequence used in this study.

Genes	Organisms	Forward Primer	Reverse Primer
*Slc3a2*	Mus musculus	TGATGAATGCACCCTTGTACTTG	GCTCCCCAGTGAAAGTGGA
*Tln1*	Mus musculus	CCTGCCGCATGATTCGTGA	TCGGAGCATGTAGTAGTCCAAA
*Capzb*	Mus musculus	CTGTGAGTGACTGTTCCCCAC	GATTTGTCTGCAAACGTCTGC
*Flna*	Mus musculus	GGCTACGGTGGGCTTAGTC	GTGGGACAGTAGGTGACCCT
*Rpn1*	Mus musculus	GCTCCACATCACGAGCCAG	CAGTTTCCACAACGACCGAGA
*β-actin*	Mus musculus	CCACCATGTACCCAGGCATT	GATTTGTCTGCAAACGTCTGC

## Data Availability

The datasets used in this paper are available online, as described in Section 4. Proteomics data are currently unpublished. Details are available upon request from the corresponding author.

## Supplementary Materials

The following supporting information can be downloaded at https://www.mdpi.com/article/10.3390/ijms252413506/s1.

## Abbreviations

AUC	Area under the curve
BP	Biological Processes
CC	Cellular Components
CeRNA	Competing endogenous RNA
CMap	Connectivity Map
CON	Control
DEGs	Differentially expressed genes
DRGs	Disulfidptosis-related genes
ECM	Extracellular matrix
ER	Endoplasmic reticulum
FDR	False discovery rate
FC	Fold change
FLNA	Filamin A
IQR	Interquartile range
GGQLT	Ge Gen Qin Lian Decoction
GLM	Golimumab
GO	Gene Ontology
GYS1	Glycogen synthase 1
IBD	Increasing inflammatory bowel disease
IFX	Infliximab
KEGG	Kyoto Encyclopedia of Genes and Genomes
LASSO	Least absolute shrinkage and selection operator
MF	Molecular Functions
NADPH	Nicotinamide adenine dinucleotide phosphate
PCA	Principal component analysis
ROS	Reactive oxygen species
SLC3A2	Solute Carrier Family 3 Member 2
SVM	Support vector machine
TLN1	Talin 1
UC	Ulcerative colitis
VDZ	Vedolizumab
WGCNA	Weighted gene co-expression network analysis
