# Supplementary material for: Disulfidptosis and Its Hub Gene Slc3a2 Involved in Ulcerative Colitis Pathogenesis, Disease Progression, and Patient Responses to Biologic Therapies
Source: Int J Mol Sci. 2024 Dec 17;25(24):13506. doi: 10.3390/ijms252413506 (PMC11728241; doi:10.3390/ijms252413506)
Supplement: Supplementary file 1 [file ijms-25-13506-s001.zip › Supplementary Materials.pdf]

Supplementary Figure S1

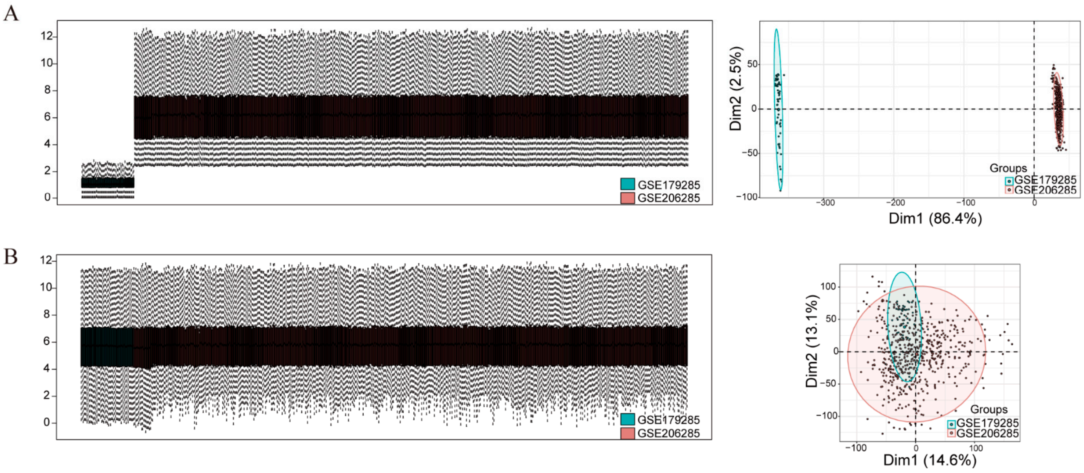

Figure S1 Data integration of the training sets

(A)Left: Batch effect analysis shows the variation between different batches of data in the integrated dataset before any normalization or debatching. The analysis highlights the systematic technical variation that could confound the results if not addressed. Right: Principal Component Analysis (PCA) was performed on the integrated datasets to assess the overall structure of the data before debatching. The PCA plot illustrates the clustering of samples based on their principal components, with each point representing a sample, and the axes reflecting the major sources of variation in the data. The plot visually indicates any batch effects, where samples from different batches may cluster together, revealing the need for further normalization or debatching. (B) Left: Batch effect analysis evaluates batch-related variations have been effectively removed after debatching the integrated datasets. This figure demonstrates that, after debatching, batch-related variation has been significantly minimized, thus enhancing the reliability and credibility of the data. Right: PCA shows the distribution of the main components in the integrated dataset after debatching. The points in the PCA plot represent individual samples, and the axes reflect the major sources of variance between them. The analysis indicates that after debatching, the samples are more tightly clustered, suggesting that the impact of batch effects has been effectively removed, further validating the success of the data integration.

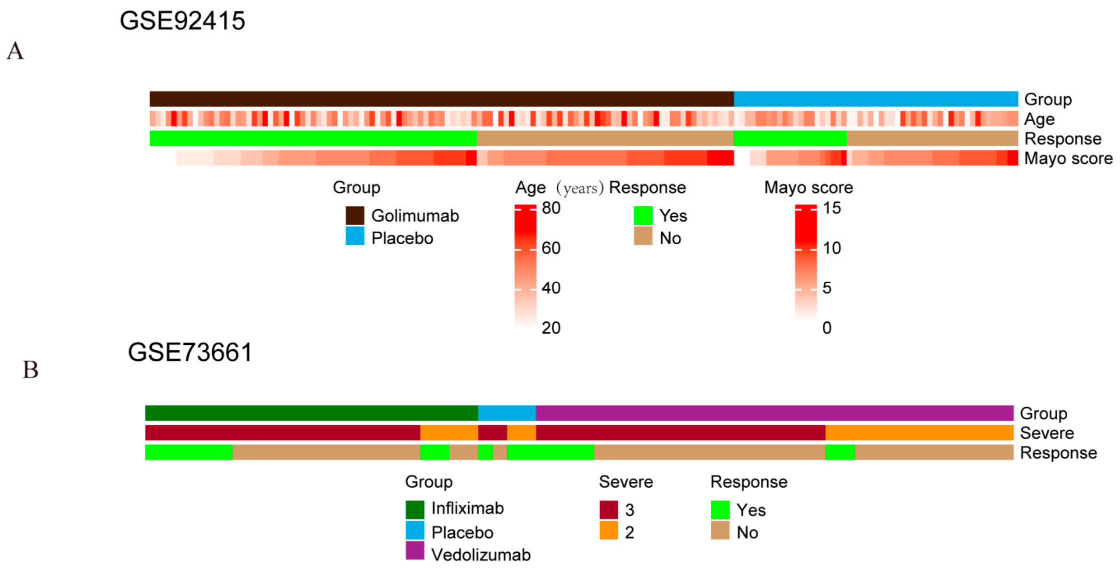

Figure S2. Heatmap of patient characteristics and disease response in the biologic-associated datasets.

(A) Heatmap of GSE92415 displaying patient characteristics and their response to golimumab treatments in UC patients. The figure represents different patient groups and their corresponding sociodemographic and clinical characteristics. Please note that gender and disease duration information are missing in this dataset.

(B) Heatmap of GSE73661 displaying patient characteristics and their response to infliximab or vedolizumab treatments in UC patients. The figure represents different patient groups and their corresponding clinical characteristics. Please note that demographic characteristics and disease duration information are missing in this dataset.

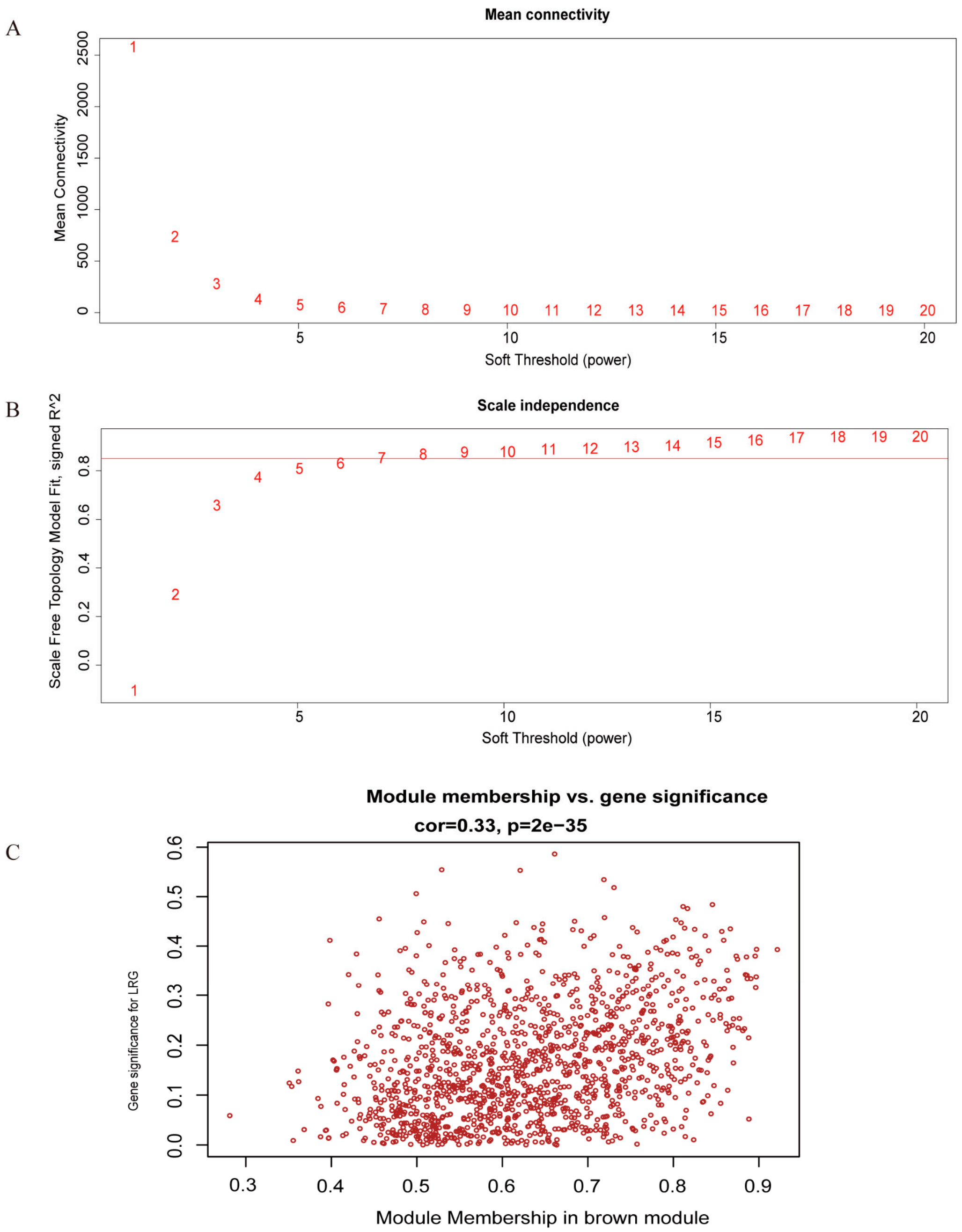

Figure S3 Relevant Parameters for WGCNA Module Identification and Selection of Module Genes

(A-B) The soft threshold for constructing the weighted gene co-expression network (WGCNA) was determined using a power value of 8, which resulted in an R^2 value of 0.85. This threshold was chosen to ensure that the gene interactions in the network adhered to a scale-free distribution, a critical characteristic for WGCNA. (C)The modules most relevant to ulcerative colitis (UC) and healthy controls were identified based on statistical significance (p-value $<$ 0.05) and a minimum correlation coefficient of 0.33. These key modules, which include UC-specific and healthy control-related gene co-expression patterns, were further analyzed to explore their potential involvement in UC pathogenesis and disease mechanisms.

Table S1. Details of the validation datasets.

Data Source	Clinical Symptoms	Patients (n)	Definition	Age (Median, Range), years	Gender Ratio (M/F)	Remarks
GSE47908	UC-associated dysplasia (UC-D)	6	The biopsy samples were taken from areas with inflammation and dysplasia, but without ulceration.	49	4/2	The dataset does not specifically disclose demographic details such as age and gender.
	Ulcerative colitis without dysplasia	39	The biopsy samples were taken from areas with inflammation, without ulceration, and without dysplasia.	37	16/23	
GSE75214	Active	74	Active disease defined as Mayo endoscopic subscore 2 for UC patients, based on endoscopic findings.	/	/	This dataset lacks demographic information
	Non-active	23	Non-active disease defined as Mayo endoscopic subscore 0-1 for UC patients, based on endoscopic findings.	/	/	
GSE87466	Limited (left-sided)	60	"Limited (left-sided) UC" refers to inflammation restricted to the left side of the colon, involving the rectum, sigmoid colon, and descending colon, and not extending beyond the splenic flexure.	41(19-77)	44/43	This dataset lacks demographic information, with only adult UC patients included and no details on sex or specific age.
	extensive (pancolitis)	27	"Extensive (pancolitis) UC" involves inflammation across the entire colon.			
GSE179285	Inflammatory	22	Endoscopically visible mucosal inflammation confirmed by histological examination.	36 (20–62)	13/9	The dataset provides biopsy samples; demographic details such as age and gender are not specifically disclosed.
	Non-inflammatory	21	Absence of visible mucosal inflammation at endoscopy and no histological inflammation confirmed by a pathologist.	38(24-64)	10/11	

Table S2. Details of the biologic-associated datasets.

Data Source	Biologic agents	Patient Diagnosis	Efficacy Assessment	Age (Median, Range), years	Gender Ratio (M/F)	Remarks
GSE92415	Golimumab(GLM)	Eligible patients had an established diagnosis of UC and moderate-to-severe disease activity, defined as a Mayo score of 6- 12, with an endoscopic subscore 2	Clinical response was defined as a decrease from baseline in the Mayo score 30% and 3 points, accompanied by either a rectal bleeding subscore of 0 or 1 or a decrease from baseline in the rectal bleeding subscore 1. Clinical remission was defined as a Mayo score 2 points, with no individual subscore >1, and mucosal healing was defined as a Mayo endoscopy subscore of 0 or 1.	/	/	The dataset has 109 samples, but the gender and age are not disclosed.
GSE73661	Infliximab (IFX)	Eligible patients had an established diagnosis of UC and moderate-to-severe disease	Histological mucosal healing was defined as a grade 0 or 1 on the Geboes score,5 and endoscopic mucosal healing was defined as a Mayo endoscopic subscore of 0 or 1.6	41.3(31.1-49.6)	13/10	The dataset information does not disclose specific gender and age.
	Vedolizumab (VDZ)			40.5(32-49.4)	21/20	

Table S6: The correlation analysis of candidate genes

CON

genes	CAPZB	FLNA	RPN1	SLC3A2	SLC7A11	TLN1
CAPZB	1	-0.30403	0.280871	-0.35474	-0.07535	0.240461
FLNA	-0.30403	1	-0.10554	0.243199	-0.21247	0.248917
RPN1	0.280871	-0.10554	1	0.003671	0.138992	0.142293
SLC3A2	-0.35474	0.243199	0.003671	1	0.124711	0.165269
SLC7A11	-0.07535	-0.21247	0.138992	0.124711	1	0.317806
TLN1	0.240461	0.248917	0.142293	0.165269	0.317806	1

UC

genes	CAPZB	FLNA	RPN1	SLC3A2	SLC7A11	TLN1
CAPZB	1	0.239512	0.246841	0.221933	-0.02614	0.488156
FLNA	0.239512	1	0.029468	0.309516	0.089788	0.667598
RPN1	0.246841	0.029468	1	0.372018	0.243364	0.186484
SLC3A2	0.221933	0.309516	0.372018	1	0.407754	0.430081
SLC7A11	-0.02614	0.089788	0.243364	0.407754	1	0.158458
TLN1	0.488156	0.667598	0.186484	0.430081	0.158458	1

Gene_1	Gene_2	cor	p.value
SLC7A11	SLC3A2	0.435388	1.84E-30
SLC7A11	FLNA	0.156795	8.60E-05
SLC7A11	CAPZB	0.05864	0.144075
SLC7A11	TLN1	0.211876	9.59E-08
SLC7A11	RPN1	0.246569	4.56E-10
SLC3A2	FLNA	0.404659	3.30E-26
SLC3A2	CAPZB	0.330811	1.19E-17
SLC3A2	TLN1	0.495365	4.00E-40
SLC3A2	RPN1	0.398748	1.94E-25
FLNA	CAPZB	0.383207	1.72E-23
FLNA	TLN1	0.702122	8.25E-94
FLNA	RPN1	0.150485	1.65E-04
CAPZB	TLN1	0.571084	2.01E-55
CAPZB	RPN1	0.35575	2.70E-20
TLN1	RPN1	0.262485	2.93E-11

Table S7 The list of characterized genes screened by least absolute shrinkage and selection operator

Number	Lasso_fea	myCoefs.x
1	SLC7A11	-81.1888931
2	SLC3A2 .	-0.1628772

3	FLNA	2.7702103
4	CAPZB	2.4240064
5	TLN1	-1.5043570
6	RPN1	6.3471007

Table S8 The list of characterized genes screened by support vector machines

Order: "CAPZB" "RPN1" "FLNA" "TLN1" "SLC3A2" "SLC7A11"

Variables	Accuracy	Kappa	AccuracySD	KappaSD
1	0.9199	0.1959	0.008143	0.1813
2	0.9336	0.3789	0.027554	0.2491
3	0.9405	0.4837	0.026242	0.2997
4	0.9496	0.5403	0.022429	0.3177
5	0.9496	0.6065	0.034103	0.2918
6	0.9702	0.7726	0.23879	0.1930

Table S9 ROC curve analysis of characterized genes in the training set

30% of the training set

CON (n=14) vs. UC (n=171)

GROUP	SLC3A2	FLNA	CAPZB	TLN1	RPN1
CON	4.948654	6.131563	8.523157	7.530932	8.538359
CON	4.989055	6.056318	8.353344	7.621745	8.726774
CON	4.652285	6.30583	8.256572	7.7539	8.268156
CON	5.075126	6.501036	8.665213	7.756741	9.115709
CON	4.880734	5.984107	8.469818	7.160623	8.589304
CON	5.6826	6.512093	8.284547	7.764676	8.783825
CON	4.992861	5.738998	8.497903	7.536907	8.445519
CON	5.02565	5.416296	8.467968	6.845372	8.732563
CON	5.4845	5.925398	8.30425	6.568816	8.749931
CON	4.996667	6.396046	8.59609	7.015341	8.95382
CON	4.885516	5.42246	8.063354	7.085288	8.867296
CON	5.666401	6.122366	8.499209	7.448739	8.499307
CON	5.027211	6.524911	8.326674	7.387413	8.550148
CON	4.806959	6.477455	8.337668	7.570509	8.615619
UC	5.722041	6.946777	9.03832	8.699868	9.039965
UC	5.640752	8.13827	9.077072	8.943802	8.836077
UC	5.680177	7.28034	9.127146	8.62904	9.114489
UC	5.497495	7.171827	8.691291	8.403132	8.55377
UC	5.950686	7.300399	9.027217	8.557917	9.069017
UC	5.204249	6.911454	8.72754	8.12354	8.347039
UC	5.832802	7.363609	9.242858	8.133826	9.163225
UC	5.162287	7.427406	9.194962	8.136471	9.270274
UC	5.678518	7.093451	8.868942	8.167428	9.207434

UC	5.457388	8.122321	9.214774	8.215039	9.244801
UC	6.309315	8.137781	9.229796	8.713877	9.313957
UC	5.905991	7.739051	9.002398	8.730923	9.195118
UC	5.28466	8.231714	8.879283	8.546945	8.965862
UC	6.254569	6.92212	8.846409	8.32378	9.225644
UC	5.432893	7.754511	9.090026	8.56017	8.866286
UC	5.930095	7.466447	9.029067	8.378053	9.319325
UC	5.442847	7.182101	8.759761	7.708559	9.032387
UC	5.34731	7.048343	9.170143	8.366689	9.067649
UC	5.360972	7.344235	8.958421	8.059764	8.962389
UC	5.692961	8.500209	9.008494	8.708587	9.020808
UC	5.439724	7.518991	8.997282	8.394119	9.136594
UC	5.830069	7.382493	9.111253	8.401467	9.054596
UC	5.908431	7.982496	8.912919	8.867585	9.179856
UC	5.945319	7.219773	9.244273	8.296742	8.992282
UC	6.268719	7.393844	9.114192	8.356207	9.290694
UC	5.401763	8.219288	9.132262	8.40754	9.338693
UC	5.630798	6.68934	9.14957	8.018815	9.155541
UC	5.959956	8.462733	8.998697	8.919016	8.806604
UC	6.010701	7.565567	8.698693	8.775204	9.108595
UC	5.537213	6.951963	8.791111	7.934761	8.483877
UC	5.165507	6.749614	8.177496	7.502245	8.800288
UC	5.882375	7.38719	9.18473	8.746598	9.261537
UC	5.483736	7.631516	9.168075	8.454956	8.976072
UC	5.697352	7.573297	9.020141	8.654217	8.755658
UC	5.60201	7.695215	8.785777	8.603765	9.090174
UC	5.928241	7.950793	8.893108	8.938707	9.041228
UC	5.405569	7.169772	9.026128	8.285574	9.182592
UC	5.709453	6.95304	9.103851	8.355913	9.054175
UC	5.770932	8.409308	9.035599	8.981322	8.842919
UC	5.581908	6.853528	9.026237	8.038408	9.253117
UC	5.786546	7.448639	9.025366	8.47592	9.020703
UC	6.677898	8.145511	9.055301	8.764036	9.486794
UC	5.868909	8.219973	9.19518	9.276785	9.103437
UC	5.969032	8.131616	9.058023	8.845445	8.894285
UC	5.650901	6.697265	9.021774	8.281949	8.838077
UC	5.134377	6.244915	8.395206	7.691023	8.894601
UC	6.030121	7.168696	8.86437	8.272642	9.223328
UC	5.422744	7.496193	9.089917	8.403426	9.04586
UC	6.345617	7.273785	8.88723	8.83604	9.014808
UC	5.778836	7.207346	9.340392	8.969371	8.90839
UC	5.942196	7.355488	9.05922	8.784216	9.219118
UC	5.870958	7.21126	9.051818	8.588972	9.01144

UC	6.260522	7.35265	8.893325	8.775008	9.114805
UC	5.15975	7.041298	8.73799	8.402544	9.342693
UC	5.662807	7.141592	8.899639	8.583192	8.830603
UC	5.828215	7.05441	9.074025	8.612679	9.278379
UC	6.259253	8.209014	8.94427	8.799793	9.274379
UC	5.266704	7.002746	9.002725	7.890187	8.658398
UC	5.492811	7.383081	8.816474	8.499628	9.295852
UC	5.549216	7.863318	8.844014	8.639032	8.644188
UC	4.577941	6.400004	8.348507	6.9652	8.901338
UC	5.959859	8.036704	8.979321	8.113939	8.562506
UC	5.537213	7.97457	9.290537	8.871699	8.971546
UC	5.572442	5.708416	9.015025	7.85678	8.855655
UC	5.597717	7.193452	9.354652	8.028513	9.047018
UC	5.341553	6.454212	9.092748	8.484541	8.944916
UC	5.264362	7.343648	8.911287	8.12305	8.858918
UC	6.067204	8.122321	9.106572	9.177742	8.793657
UC	5.821677	7.073784	8.938609	8.40323	9.393639
UC	6.078329	7.821341	9.241443	8.764525	9.236907
UC	5.648949	6.630925	8.89289	8.450841	8.960073
UC	5.818554	8.138955	9.055955	8.76815	9.096595
UC	6.012165	7.769971	9.09351	8.776477	9.045965
UC	5.539262	7.320556	8.992601	8.276463	9.257538
UC	6.00026	7.546389	9.095142	8.620909	8.984177
UC	5.81426	7.487386	8.918253	8.624435	9.187329
UC	5.353458	7.552651	8.935561	8.918527	8.679766
UC	5.972545	7.106465	9.022645	8.478271	9.008808
UC	5.578199	7.918504	8.957985	8.702611	8.806814
UC	5.911261	7.556075	8.845756	8.74072	8.959126
UC	5.659977	6.584349	8.529315	7.37489	8.781657
UC	5.561805	6.972707	9.34061	8.457993	9.307115
UC	6.000064	7.26537	9.296197	8.899325	9.306168
UC	5.597229	7.906762	8.908021	8.823011	9.254485
UC	5.701353	7.684158	8.925547	8.478369	9.012492
UC	5.451728	7.174078	8.776851	8.106298	9.341956
UC	5.624065	7.149811	9.160891	8.644714	8.844813
UC	5.772005	7.967721	9.057152	8.938903	9.132594
UC	6.289212	7.926234	8.958965	8.829476	9.306799
UC	5.338332	7.62907	9.121921	8.528136	8.848076
UC	6.107702	8.244337	9.202147	9.042159	8.65503
UC	5.923166	6.826718	8.961577	8.361791	9.07007
UC	6.066423	8.23739	9.044416	9.251706	8.990809
UC	5.756196	8.1187	9.158496	8.593478	8.972704
UC	5.807527	6.739731	8.714695	7.350986	8.587769

UC	5.417475	8.898645	9.01459	8.811255	8.43451
UC	5.931754	6.642373	8.990097	8.30693	9.088701
UC	5.582883	7.109302	8.912484	8.749243	8.681976
UC	6.043978	7.407934	9.184403	8.147443	9.103016
UC	5.560731	6.421335	9.092094	8.244624	9.024597
UC	5.822555	7.842769	9.094816	8.969566	9.223223
UC	6.06574	7.736898	9.310566	8.725535	9.033755
UC	6.160984	6.888754	9.030483	8.290276	9.391744
UC	5.452996	7.571438	9.013175	8.727495	8.891864
UC	5.674127	7.323687	9.155013	8.316335	8.967441
UC	5.759124	7.115663	9.038429	8.11247	9.340482
UC	5.484517	7.568502	9.248083	8.735136	8.631767
UC	5.891744	7.901282	8.836939	9.307938	9.030281
UC	5.373951	7.640029	9.06118	8.569281	8.921548
UC	5.55351	7.024371	8.985743	8.287729	9.112068
UC	5.611769	6.757148	9.05541	8.007941	8.848603
UC	5.912237	8.112438	9.30643	8.958496	9.122594
UC	6.002114	7.421143	9.002289	8.69066	8.922706
UC	5.439529	7.364587	9.024278	8.606606	8.793341
UC	5.960249	7.203921	8.979321	8.549786	9.273748
UC	5.421671	7.477895	8.747895	8.267646	8.770816
UC	5.830752	7.362924	8.826489	8.034195	9.012598
UC	5.924533	7.783474	8.912811	8.536659	9.07449
UC	6.185771	7.126817	9.106137	7.903314	8.911443
UC	6.174549	7.484744	8.950257	9.007479	9.060596
UC	5.69218	7.119576	9.112015	8.282537	9.159962
UC	5.815724	7.512827	8.745609	8.484933	9.058807
UC	6.207923	8.191597	9.235783	8.854359	9.404059
UC	5.782545	7.825255	9.102218	8.742287	9.334693
UC	6.16762	7.620949	8.905408	8.907261	9.012177
UC	6.322684	6.723195	9.391663	8.344157	9.473005
UC	5.829093	7.457347	9.053778	8.514714	9.128384
UC	5.769468	6.814976	9.19039	8.728572	9.241117
UC	5.368779	7.285428	8.886577	8.456523	8.783447
UC	6.091893	7.382591	9.196051	8.858376	8.973757
UC	5.723798	6.022507	8.954937	8.134904	8.955863
UC	5.866957	7.243158	9.182226	8.246584	9.234801
UC	6.037635	7.077208	8.905953	8.711918	9.195013
UC	6.218657	7.37946	9.041259	8.370902	9.129752
UC	5.558487	6.286403	8.560447	7.443074	8.536612
UC	6.008945	7.337581	8.825727	8.144504	9.172909
UC	5.648461	6.862335	8.85109	8.538814	9.01344
UC	5.712771	8.745317	8.978015	8.929989	9.04586

UC	6.065252	8.100403	8.782947	8.811255	8.721133
UC	5.427136	6.799321	9.047573	8.180849	8.599452
UC	5.670516	7.656663	9.035708	8.513343	8.830182
UC	6.026901	7.597563	9.004031	8.191429	8.940284
UC	6.407487	7.229753	8.862628	9.032166	9.109647
UC	5.852807	6.939047	8.890386	8.376975	9.131857
UC	5.375122	7.502259	9.22272	8.867781	8.863023
UC	5.744291	7.834746	8.958203	8.669989	9.20175
UC	5.985231	8.44424	8.989444	9.060576	8.96081
UC	6.073742	8.384553	9.166442	8.892076	9.096385
UC	5.609037	8.274572	9.04496	8.629432	8.824182
UC	6.344348	7.694139	9.289666	8.579175	9.108279
UC	6.010604	7.636017	9.055628	8.754043	9.371324
UC	6.289212	8.478389	9.171232	9.017961	9.15733
UC	5.368291	6.843059	8.975075	8.340238	8.874076
UC	5.858564	7.398051	9.327983	8.666952	9.135541
UC	6.088965	7.78954	9.017093	8.421647	9.481215
UC	5.667979	7.696291	9.233388	8.814194	8.782815
UC	5.710819	7.213608	9.292278	8.708783	8.929127
UC	5.538287	6.926621	9.023842	8.129515	9.188803
UC	5.830069	7.402259	8.936105	8.501195	9.181856
UC	5.410839	7.741791	9.0602	8.656764	8.908706
UC	5.417475	6.790319	9.189193	9.236032	8.778394
UC	6.400461	8.488173	8.995322	9.250922	8.843024
UC	5.126863	7.589539	9.1106	8.398136	8.715344
UC	5.389272	7.919874	9.015134	8.796756	8.852708
UC	5.854466	7.607837	9.005555	8.495317	8.757027
UC	5.64368	7.78817	8.892346	8.19672	8.692607
UC	5.380587	6.621825	8.842055	8.02724	8.559033
UC	5.838559	6.989635	8.955808	8.246682	9.147541
UC	5.682616	7.026034	9.23317	8.423509	8.883128
UC	6.20968	8.370952	9.370654	9.025701	9.049333
UC	5.677444	6.705778	8.724383	7.950631	9.191645

Table S10 ROC curve analysis of characterized genes in the validation set

GSE48958

CON (n=8) vs. UC (n=13)

	Group	SLC3A2	FLNA	CAPZB	TLN1	RPN1
GSM1187602	CON	335.4	375.3	656	374.6	771.8
GSM1187603	CON	296.4	306.9	664.8	425.4	776.1
GSM1187604	CON	405.5	404.4	812	415.5	821.5
GSM1187605	CON	318.4	600.9	547	444.5	845.6
GSM1187606	CON	289.9	283.5	680.4	413.5	922.1

GSM1187607	CON	349.1	472.5	661.8	435.5	800
GSM1187608	CON	361.6	585.4	708.5	489.1	775.9
GSM1187609	CON	291.7	413.9	714	350	775.1
GSM1187610	UC	319.1	470.1	705.4	507.1	1205.6
GSM1187611	UC	403.2	816.4	749.8	751.8	1215.2
GSM1187612	UC	328.7	1175	702.4	874.2	952.4
GSM1187613	UC	497.2	1041.5	938.6	693.9	1240.9
GSM1187614	UC	312.8	653.5	694.2	579.3	1042.2
GSM1187615	UC	401.6	1073.2	753.5	696.6	1169.7
GSM1187616	UC	409	925.5	773	575.8	1114.1
GSM1187617	UC	277.8	1763.2	768	634.7	818.1
GSM1187618	UC	377.1	2312.2	758.7	699.1	748.2
GSM1187619	UC	372.1	464.8	859.1	499.5	707
GSM1187620	UC	302.2	871.1	891	411.6	849.1
GSM1187621	UC	221	685.9	601.8	432.3	686.5
GSM1187622	UC	187.2	569	561.8	382.1	799.9

GSE47908
CON (n=15) vs. UC (n=39)

	Group	SLC3A2	FLNA	CAPZB	TLN1	RPN1
GSM1162227	CON	9.131669	8.226585	11.26175	7.038992	10.61849
GSM1162228	CON	9.011318	8.321247	11.37071	7.176288	10.57536
GSM1162229	CON	8.860233	8.238518	11.18582	7.018518	10.48336
GSM1162230	CON	8.412947	7.865442	11.3233	6.945052	10.68375
GSM1162231	CON	8.748113	8.737835	11.22399	7.14039	10.68816
GSM1162232	CON	8.776253	8.073125	11.33502	7.319031	10.70227
GSM1162233	CON	8.882845	8.533315	11.55362	7.300484	10.93252
GSM1162234	CON	8.504066	8.284764	11.54287	7.239213	10.5999
GSM1162235	CON	8.754362	8.055657	11.49268	7.038271	10.74893
GSM1162236	CON	8.796644	8.580036	11.6154	7.363208	10.94981
GSM1162237	CON	8.930279	8.324376	11.6126	7.014281	10.93905
GSM1162238	CON	8.866042	8.084216	11.54242	7.485095	10.63108
GSM1162239	CON	8.296365	8.029131	11.63157	7.245637	10.36123
GSM1162240	CON	8.532453	8.464994	11.6007	7.185706	10.90847
GSM1162241	CON	8.675191	8.568873	11.65357	7.18445	10.74039
GSM1162248	UC	8.898334	9.864227	11.62718	7.981799	10.92876
GSM1162249	UC	9.412531	9.396225	11.59043	7.423591	11.29496
GSM1162250	UC	9.00836	8.931355	11.45954	7.541224	11.29673
GSM1162251	UC	8.792978	8.169252	11.4514	7.230416	10.86445
GSM1162252	UC	8.794724	8.793233	11.64714	7.29281	11.12622
GSM1162253	UC	9.030668	8.622551	11.61479	7.358731	11.38375
GSM1162254	UC	8.984016	8.793324	11.74603	7.381287	11.08781

GSM1162255	UC	8.97507	8.769752	11.68686	7.315748	11.13603
GSM1162256	UC	8.910359	8.212943	11.54945	7.128105	11.25831
GSM1162257	UC	8.771811	10.28561	11.65651	7.965071	10.90348
GSM1162258	UC	8.826324	8.910309	11.64898	7.25783	11.2378
GSM1162259	UC	8.904055	8.516325	11.61658	7.281523	11.27189
GSM1162260	UC	9.067191	8.991233	11.5376	7.484366	11.26788
GSM1162261	UC	8.793033	8.524614	11.32654	7.214954	11.17093
GSM1162262	UC	8.662579	8.178075	11.67997	7.224043	10.94871
GSM1162263	UC	8.965947	8.79495	11.35439	7.473204	11.1732
GSM1162264	UC	8.796431	8.918651	11.46443	7.337929	11.28948
GSM1162265	UC	9.732595	8.51586	11.5543	7.325456	11.13345
GSM1162266	UC	8.887101	8.852807	11.62727	7.675143	11.13912
GSM1162267	UC	8.755009	9.171434	11.64256	7.244073	11.15244
GSM1162268	UC	8.224177	8.356634	11.8421	7.11221	11.05711
GSM1162269	UC	8.684456	8.552344	11.94846	7.051851	11.01801
GSM1162270	UC	8.702272	9.168983	11.60906	7.362919	10.96151
GSM1162271	UC	8.586679	8.773398	11.49996	6.799718	11.24369
GSM1162272	UC	8.215692	8.023093	11.77614	7.136567	11.089
GSM1162273	UC	8.736936	8.558723	11.65054	7.378963	11.08679
GSM1162274	UC	8.218298	9.151066	11.79294	7.375837	11.01223
GSM1162275	UC	8.384969	8.035774	11.76724	6.799783	11.11656
GSM1162276	UC	8.464233	9.064257	11.75363	7.29287	11.29738
GSM1162277	UC	8.568596	9.340981	11.75836	7.43496	11.2858
GSM1162278	UC	8.437691	9.327888	11.72602	6.981688	11.29269
GSM1162279	UC	8.161841	8.447549	11.9946	6.62411	10.19841
GSM1162280	UC	8.650028	10.04749	11.65897	7.422622	10.93141
GSM1162281	UC	8.852804	9.166095	11.48423	7.218472	11.38233
GSM1162282	UC	8.843728	8.378216	11.83239	7.211695	10.94965
GSM1162283	UC	8.734751	8.846455	11.76143	7.2316	11.28559
GSM1162284	UC	8.212879	8.134936	12.01972	7.012372	10.83633
GSM1162285	UC	8.580025	9.084016	11.72318	8.535022	10.99056
GSM1162286	UC	9.16565	8.145035	11.48714	7.183966	11.01136

GSE92415
CON (n=21) vs. UC (n=87)

	group	SLC3A2	FLNA	CAPZB	TLN1	RPN1
GSM2429455	CON	7.5684	8.1968	11.2113	10.3621	10.579
GSM2429456	CON	7.6531	7.5726	10.686	8.8405	10.0694
GSM2429457	CON	7.0386	7.7266	10.9576	9.9274	10.0765
GSM2429458	CON	7.5917	7.525	10.9629	9.554	9.857
GSM2429459	CON	7.411	7.8744	10.8967	9.7584	10.134
GSM2429460	CON	7.3858	7.9543	10.9327	10.3109	10.2876

GSM2429461	CON	7.7848	8.872	11.1771	10.3683	10.7567
GSM2429462	CON	8.3259	8.2974	11.0238	10.6119	10.2108
GSM2429463	CON	8.2932	10.3596	10.907	10.6817	10.0945
GSM2429464	CON	7.9394	8.7856	11.191	10.2687	10.0929
GSM2429465	CON	6.93	7.0473	10.4498	9.0109	9.8904
GSM2429466	CON	6.8586	6.0654	10.4157	9.3764	9.6983
GSM2429467	CON	7.0953	7.6369	11.1992	9.8474	10.3593
GSM2429468	CON	6.5089	6.8373	9.5342	9.2208	9.3703
GSM2429469	CON	7.1763	7.4748	10.1858	9.396	9.7079
GSM2429470	CON	7.5323	6.7933	10.544	9.1759	10.1027
GSM2429471	CON	7.1885	7.7895	11.0914	9.5951	10.1739
GSM2429472	CON	8.0021	9.2016	11.0224	10.2401	10.3859
GSM2429473	CON	7.149	7.1592	11.21	9.5548	10.4994
GSM2429474	CON	7.1139	7.7577	11.1046	10.0136	10.2069
GSM2429475	CON	7.1926	6.6145	10.6335	8.7631	10.5468
GSM2429346	UC	8.1188	9.4408	11.0243	10.5522	11.2667
GSM2429347	UC	8.1779	9.5144	10.9649	10.4482	11.1349
GSM2429348	UC	7.6257	7.7856	11.0752	9.1458	10.7508
GSM2429349	UC	7.6951	8.5139	11.0278	10.2639	10.7911
GSM2429350	UC	7.5618	7.5573	10.9399	9.311	10.6673
GSM2429351	UC	7.1954	8.6621	11.1198	10.5193	10.5777
GSM2429352	UC	7.911	8.6831	10.9763	10.3112	10.8779
GSM2429353	UC	7.535	7.8644	11.0422	9.9686	10.8215
GSM2429354	UC	7.748	8.4737	11.1945	10.341	10.8474
GSM2429355	UC	7.7231	8.1277	11.2248	10.0776	11.0543
GSM2429356	UC	8.2845	9.1834	11.1586	10.6696	11.1067
GSM2429357	UC	7.2597	8.7093	11.1458	9.9391	10.7966
GSM2429358	UC	7.7873	8.6837	11.2592	10.2885	10.8625
GSM2429359	UC	7.6319	8.8959	11.1259	10.3725	10.5723
GSM2429360	UC	7.5817	7.812	11.2825	9.7935	10.5909
GSM2429361	UC	8.1157	9.0434	11.1753	10.5429	10.6728
GSM2429362	UC	8.0624	8.8087	11.0647	10.2849	11.0296
GSM2429363	UC	7.6557	9.0257	11.2283	10.3744	10.5098
GSM2429364	UC	7.9226	8.6858	11.233	10.4674	10.1504
GSM2429365	UC	8.0936	10.0764	11.2798	10.9987	10.2442
GSM2429366	UC	8.2073	9.8367	10.9834	10.9548	10.3132
GSM2429367	UC	8.0583	8.7489	11.1463	10.5255	10.0853
GSM2429368	UC	8.2181	10.1152	11.1128	10.7481	9.7804
GSM2429369	UC	8.1889	8.923	11.0931	10.5601	10.5621
GSM2429370	UC	7.5676	8.312	11.1319	10.1407	9.9881
GSM2429371	UC	8.1448	9.8421	10.7155	10.7064	10.5775
GSM2429372	UC	8.9792	10.0276	10.9909	11.0062	10.7082
GSM2429373	UC	7.7795	8.5498	11.2046	10.116	9.9348

GSM2429374	UC	7.8537	8.6081	10.9012	10.3915	10.4874
GSM2429375	UC	7.835	8.3558	10.9056	10.1716	10.3573
GSM2429376	UC	7.6327	9.7434	11.0389	10.6473	9.9888
GSM2429377	UC	7.3167	8.1471	10.861	10.0883	9.9714
GSM2429378	UC	6.8973	8.4184	10.3601	10.115	9.9458
GSM2429379	UC	7.3035	7.7118	10.4244	9.9364	10.1348
GSM2429380	UC	7.5503	8.5336	10.6048	10.1648	10.4253
GSM2429381	UC	7.7214	8.2101	10.7024	10.0871	10.631
GSM2429382	UC	6.8684	7.8335	10.4702	9.8182	10.2042
GSM2429383	UC	7.2991	8.899	11.2481	10.5559	10.1868
GSM2429384	UC	7.1451	7.9497	10.3132	9.0629	10.5328
GSM2429385	UC	7.7664	8.4924	11.1424	10.2171	10.8822
GSM2429386	UC	7.4645	9.0357	11.3152	10.7168	10.2625
GSM2429387	UC	7.5811	9.06	11.1919	10.552	10.5337
GSM2429388	UC	8.0695	10.0767	11.0505	10.9053	10.8457
GSM2429389	UC	7.2659	9.4128	11.2487	10.4106	10.38
GSM2429390	UC	7.4017	8.2826	11.1801	9.7601	10.5073
GSM2429391	UC	6.9751	8.1114	10.9621	10.1598	10.2845
GSM2429392	UC	7.117	8.8224	11.0899	9.917	10.0994
GSM2429393	UC	8.1229	9.8075	11.2484	10.6274	11.2096
GSM2429394	UC	6.9642	7.6764	10.6664	9.6936	9.7396
GSM2429395	UC	7.1492	8.2604	11.3542	10.0675	10.7662
GSM2429396	UC	7.5268	8.7513	11.1518	10.4234	10.6624
GSM2429397	UC	7.8154	9.407	11.0264	10.7549	10.6898
GSM2429398	UC	8.1488	9.7724	10.8462	10.8873	9.6278
GSM2429399	UC	7.9556	8.2279	11.0506	9.6791	10.1696
GSM2429400	UC	7.3988	9.2796	11.0151	10.2542	9.9859
GSM2429401	UC	6.5321	7.3462	10.3103	9.7709	9.9286
GSM2429402	UC	7.3786	8.5381	10.5864	9.8264	10.1402
GSM2429403	UC	7.919	8.0161	10.6898	9.507	10.8586
GSM2429404	UC	7.801	8.4966	11.2471	10.2565	10.4762
GSM2429476	UC	7.7246	8.6228	11.2022	10.5003	10.9504
GSM2429477	UC	7.6896	8.2155	11.2075	10.1627	10.9277
GSM2429478	UC	7.4986	9.3415	11.0379	10.4173	10.8384
GSM2429479	UC	7.3874	9.1223	11.0793	10.3702	10.4841
GSM2429480	UC	7.8238	9.4889	11.2143	10.655	10.0765
GSM2429481	UC	7.6037	8.4656	11.0584	10.3405	10.5268
GSM2429482	UC	7.948	7.7743	11.1529	9.8256	10.7135
GSM2429483	UC	7.4852	9.2394	11.1133	10.2582	10.4646
GSM2429484	UC	8.1467	9.0823	11.3392	10.5005	11.061
GSM2429485	UC	8.0521	9.3134	11.1304	10.5394	10.9124
GSM2429486	UC	8.1165	9.5991	11.0905	10.7752	10.2362
GSM2429487	UC	7.9412	9.8097	11.204	10.9727	10.1063

GSM2429488	UC	7.5569	9.0456	10.9511	10.6789	9.8424
GSM2429489	UC	8.1751	9.6793	10.9737	10.8933	10.3785
GSM2429490	UC	7.6745	8.5497	10.3602	10.4606	10.4164
GSM2429491	UC	7.1041	6.6248	10.738	9.2128	10.1657
GSM2429492	UC	7.3265	8.3772	10.4523	10.1166	10.1111
GSM2429493	UC	7.3115	7.7757	10.4384	9.2606	10.2929
GSM2429494	UC	7.3971	8.5796	10.6165	10.2066	10.0469
GSM2429495	UC	7.3058	7.8887	11.2775	10.2072	10.4033
GSM2429496	UC	8.2754	8.7298	11.159	10.0906	10.9899
GSM2429497	UC	8.4034	9.6044	11.1991	10.8788	10.99
GSM2429498	UC	7.6816	8.36	11.2072	10.1525	10.4805
GSM2429499	UC	7.5659	8.7757	11.0663	10.5547	10.0658
GSM2429500	UC	7.3734	8.7524	11.2611	10.4594	10.2153
GSM2429501	UC	7.2483	8.0047	11.0746	9.4993	10.6668
GSM2429502	UC	8.2334	9.8043	11.1417	10.7355	10.5625
GSM2429503	UC	7.7711	9.3071	11.1033	10.5968	10.0403
